# New Dinuclear Macrocyclic Copper(II) Complexes as Potentially Fluorescent and Magnetic Materials

**DOI:** 10.3390/ijms24033017

**Published:** 2023-02-03

**Authors:** Magdalena Barwiolek, Dominika Jankowska, Anna Kaczmarek-Kędziera, Iwona Lakomska, Jedrzej Kobylarczyk, Robert Podgajny, Paweł Popielarski, Joanna Masternak, Maciej Witwicki, Tadeusz M. Muzioł

**Affiliations:** 1 Faculty of Chemistry, Nicolaus Copernicus University in Torun, Gagarina 11, 87-100 Torun, Poland; 2 Institute of Nuclear Physics PAN, Radzikowskiego 152, 31-342 Kraków, Poland; 3 Faculty of Chemistry, Jagiellonian University, Gronostajowa 2, 30-387 Kraków, Poland; 4 Faculty of Physics, Kazimierz Wielki University, Powstancow Wielkopolskich 2, 85-090 Bydgoszcz, Poland; 5 Institute of Chemistry, Jan Kochanowski University of Kielce, Uniwersytecka 7, 25-406 Kielce, Poland; 6 Faculty of Chemistry, University of Wrocław, Joliot Curie 14, 50-383 Wrocław, Poland

**Keywords:** oligonuclear complexes, magnetic properties, fluorescence, DFT, Hirshfeld analysis, EPR

## Abstract

Two dinuclear copper(II) complexes with macrocyclic Schiff bases **K1** and **K2** were prepared by the template reaction of (*R*)-(+)-1,1′-binaphthalene-2,2′-diamine and 2-hydroxy-5-methyl-1,3-benzenedicarboxaldehyde **K1**, or 4-*tert*-butyl-2,6-diformylphenol **K2** with copper(II) chloride dihydrate. The compounds were characterized by spectroscopic methods. X-ray crystal structure determination and DFT calculations confirmed their geometry in solution and in the solid phase. Moreover, intermolecular interactions in the crystal structure of **K2** were analyzed using 3D Hirshfeld surfaces and the related 2D fingerprint plots. The magnetic study revealed very strong antiferromagnetic Cu^II^-Cu^II^ exchange interactions, which were supported by magneto-structural correlation and DFT calculations conducted within a broken symmetry (BS) framework. Complexes **K1** and **K2** exhibited luminescent properties that may be of great importance in the search for new OLEDs. Both **K1** and **K2** complexes showed emissions in the range of 392–424 nm in solutions at various polarities. Thin materials of the studied compounds were deposited on Si(111) by the spin-coating method or by thermal vapor deposition and studied by scanning electron microscopy (SEM/EDS), atomic force microscopy (AFM), and fluorescence spectroscopy. The thermally deposited **K1** and **K2** materials showed high fluorescence intensity in the range of 318–531 nm for **K1**/Si and 326–472 nm for the **K2**/Si material, indicating that they could be used in optical devices.

## 1. Introduction

Macrocyclic metal complexes with Schiff bases are of great interest to researchers, thanks to various properties of importance in design and synthesis of functional molecular materials [1,2,3,4,5,6,7]. Complexes with optically active Schiff bases, particularly binaphthyl-based macrocycles, stand out significantly, revealing optical properties to find applications as organic compound sensors, colourimetric sensors, or fluorescent sensors for chiral recognition [8,9,10,11,12]. One of the greatest applications of these compounds is catalytic activity in various organic reactions [13,14]. Moreover, the Schiff bases and their complexes exhibit biological properties, e.g., anticancer and antibacterial activity [15,16].

A large number of donor atoms, such as O, N, S, P, and supramolecular construction, afford sufficient space for coordination atoms to create mono-, di-, and polynuclear complexes [17,18,19]. Macrocycle architecture consisting of a combination of binaphthyl groups can also show fluorescent properties. 1,1’-Binaphthyl units can be functionalized at the 2- and 2’-positions and are often chiral due to restricted rotation about the transannular bond [20]. Thus, their role in asymmetric synthesis and as a ligand in supramolecular chemistry is not to be underestimated [21]. The number of donor atoms can be changed, and thus various types of complexes can be achieved. Recently, E. Chinnaraja et al. reported the one-pot synthesis of a series the macrocyclic complexes [2+2] consisting of dialdehyde: 4-methyl-2,6-diformylphenol or 4-*tert*-butyl-2,6-diformylphenol and chiral binaphthyl amine as building blocks. Cu(II) metal salt was used in these syntheses. The obtained compounds were enantiomerically pure, showed chiroptical properties, and exhibited catalytic activity in many reactions of organic transformations [22,23,24]. Great interest was shown in the design, synthesis, and modification of complexes due to their possible impact on the field of molecular magnetism [25,26]. The study of magnetic interaction between the central Cu(II) ions in multinuclear complexes is the main purpose of magneto-structural investigations [27,28]. Considering magnetic properties, dinuclear macrocyclic complexes with Cu(II) deserve to be recognized [29,30,31]. Consequently, new compounds with fluorescent and magnetic properties that could be used in thin films are still sought for. It is also known that Schiff base complexes could create thin materials by using wet methods, e.g., dip or spin coating, as well as vapor deposition techniques. The properties of the materials depend on the presence of substituents, their electronic nature, or the presence and size of the aromatic rings. The choice of suitable substrates for designing new materials is one of the most important factors that can determine the unusual properties of the received films and their application. There are still many questions regarding the fluorescence and magnetic properties of the compounds and thin materials. The appropriate methods for creating new films should also be developed. New materials can improve crucial parameters such as life and durability of the new films, high luminescence, and designs, as well as provide new unique characteristics of the new devices such as smartphones, OLEDS, or solar batteries. Therefore, we report the synthesis, structure, and magnetic and spectroscopic properties of binuclear macrocyclic Cu(II) complexes obtained in the one-pot reaction of (R)-(+)-1,1′-binaphthalene-2,2′-diamine and 2-hydroxy-5-methyl-1,3-benzenedicarboxaldehyde **K1**, or 4-*tert*-butyl-2,6-diformylphenol **K2** with copper(II) chloride dihydrate. DFT calculations were also carried out to support the magnetic behavior and optical properties of the complexes. Hirshfeld analysis extensively shows intramolecular interactions. The new copper(II) complexes were used as precursors of thin layers in the spin coating technique and thermal vapor deposition. The morphology and composition of layers were analyzed by AFM and SEM/EDX microscopy, and the fluorescence properties of the thin materials were also studied. Both of the presented copper(II) complexes exhibited strong antiferromagnetic Cu^II^-Cu^II^ exchange interactions.

## 2. Results and Discussion

### 2.1. Dinuclear Copper(II) Complexes: Synthesis and Characterization

**Figure 1:**
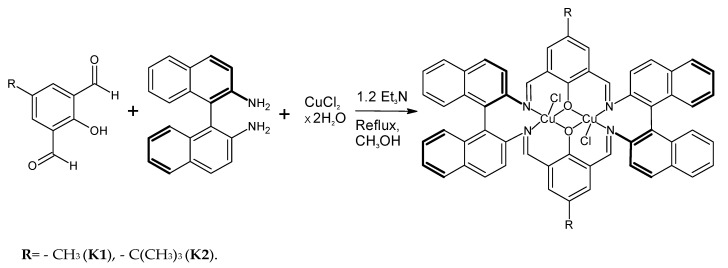
Scheme of the copper(II) complex **K1** and **K2** synthesis.

**Figure 2:**
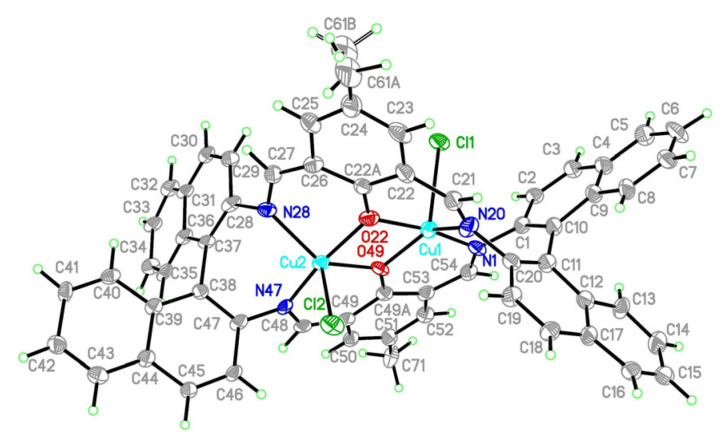
Structure of [Cu_2_Cl_2_(L’)] **K1** with a numbering scheme and thermal ellipsoids at 30% probability.

**Figure 3:**
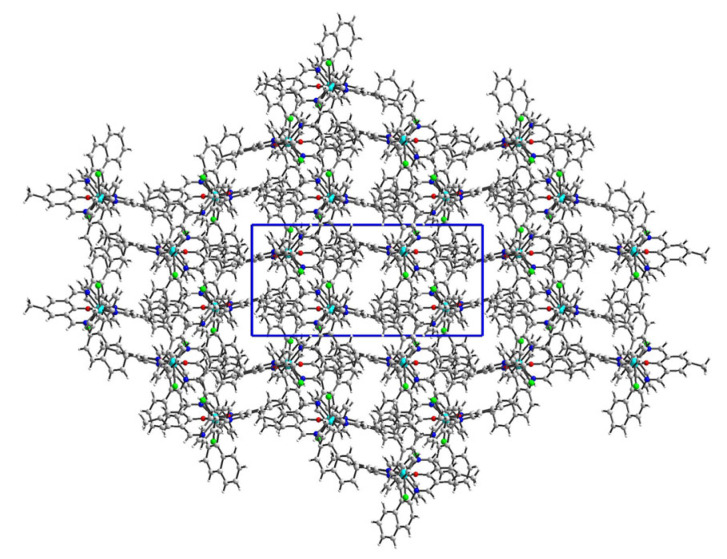
The crystal network of **K1** showed channels running along the *c* axis. The unit cell is given in blue box.

**Figure 4:**
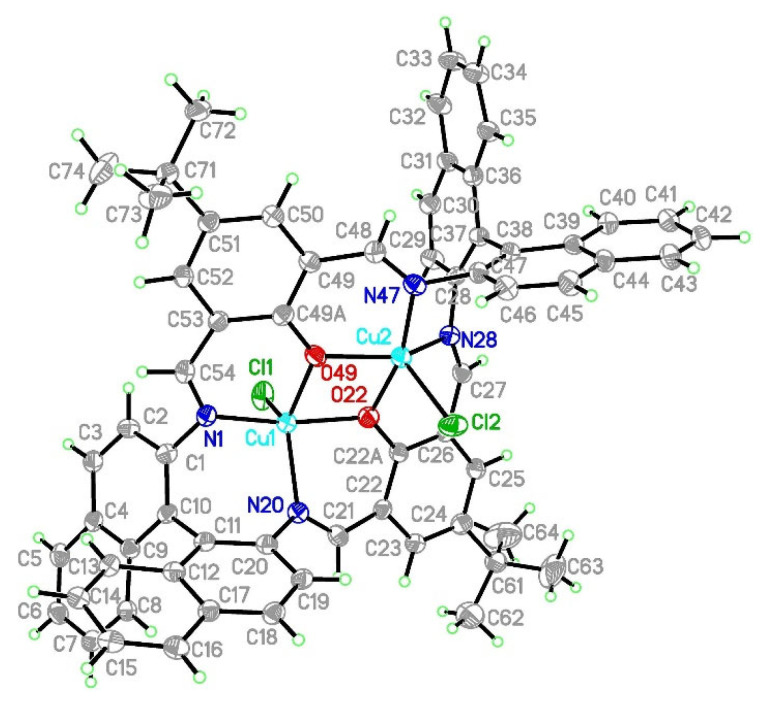
Structure of [Cu_2_Cl_2_(L)]·H_2_O **K2** with a numbering scheme and thermal ellipsoids at 30% probability.

**Figure 5:**
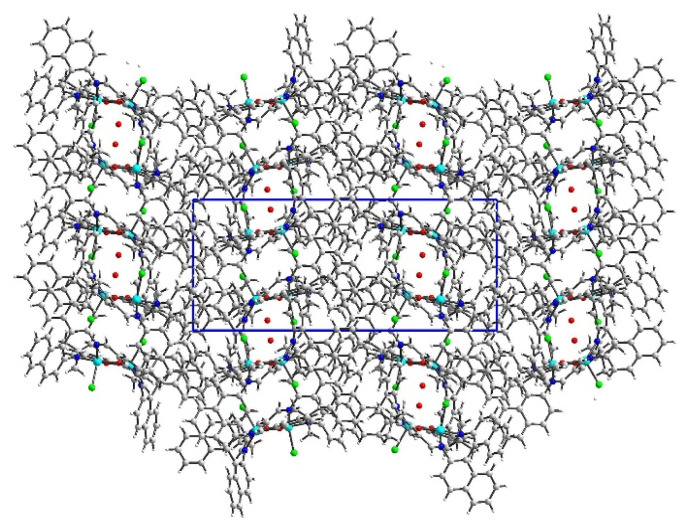
The crystal network of **K2** showing channels running along the *b* axis filled with water molecules. The unit cell is given in blue box.

Reactions of template synthesis in a molar ratio of 1:1:1 resulted in dinuclear macrocyclic copper(II) complex formation (Figure 1). The IR spectra exhibited the -C=N- stretching bands, characteristic for the Schiff bases (Figures S1 and S2). The elemental analysis and X-ray studies confirmed the purity of the obtained compounds (Figure 2, Figure 3, Figure 4 and Figure 5). The thermal stability of **K1** and **K2** complexes was studied by thermogravimetric analysis from ambient temperature to 1000 °C under air (Figures S3–S5). The final decomposition product was a mixture of copper(II) oxide and copper(I) oxide that was additionally confirmed by the XRD analysis (Figures S6 and S7). 

### 2.2. Crystal Structure Description

#### 2.2.1. Crystal Structure Description of [Cu_2_Cl_2_(L’)] **K1**

The structure of the [Cu_2_Cl_2_(L’)] (**K1**) (L’ = (R)-(+)-1,1′-binaphthalene-2,2′-diamine and 2-hydroxy-5-methyl-1,3-benzenedicarboxaldehyde) complex crystallized in the orthorhombic space group P2_1_2_1_2_1_ with all atoms in the general positions. Copper(II) cations are found in a heavily distorted pentacoordinated environment (τ_5_ = 0.47 and 0.51 for Cu1 and Cu2, respectively [32]) (Figure 2). The analysis of polyhedra carried out in SHAPE [33] showed that for Cu1, we cannot discriminate between trigonal bipyramid (S_TBPY_ =3.224) and square pyramid (S_SPY_ = 3.522), whereas for Cu2, trigonal bipyramid (S_TBPY_ =1.882, S_SPY_ = 2.859) should be selected. Coordination spheres of both copper(II) ions consist of two nitrogen atoms and two oxygen atoms from the macrocyclic ring and apical chloride anion. Both copper polyhedra share the common edge (O22 – O49) with deprotonated hydroxyl groups bridging both copper(II) ions separated by 3.109 Å. The longest bonds occurred for apical chloride anions (2.495(3) and 2.363(2) Å) (Table 1). Cu-N/O bonds ranged from 1.917(11) to 2.034(10) and from 1.923(7) to 2.069(9) Å for Cu1 and Cu2, respectively. They were similar to those found in the polymorph (1.916(2)–2.078(4) Å) with significantly larger cells and lower density [24]. This is the only structural report for copper complexed in binaphthyl macrocyclic system. Mutual orientation of C1 and C11 (69.1°) as well as C28 and C38 (75.1°) naphthyl rings imposes the chirality of the complex. The Flack parameter shows that pure enantiomer was obtained. The dihedral angle between C22a and C49a phenyl rings was 33.1°. 

In the crystal network, there are voids accounting for 1231.5 Å^3^ (22.7% of the volume cell) (Figure 3). The solvent density was poorly defined, and hence the bypass procedure was performed in Olex2 [34], resulting in a much better model. Nevertheless, the interaction model suffered from missing complex–solvent interactions. Therefore, we did not apply the Hirshfeld approach for intermolecular interactions. We limited our description only to complex–complex contacts. They were assured mainly by π–π and C–H…π interactions. The pore structure was also stabilized by a C71–H71B…Cl1[-x, 1-y, z] hydrogen bond. 

#### 2.2.2. Crystal Structure Description of [Cu_2_Cl_2_(L)]·H_2_O **K2**

The structure of the [Cu_2_Cl_2_(L″)]·H_2_O **K2** (L″ = (*R*)-(+)-1,1′-binaphthalene-2,2′-diamine and 4-*tert*-butyl-2,6-diformylphenol) complex crystallized in the chiral orthorhombic P2_1_2_1_2_1_ space group with all atoms in general positions and the whole molecule given by the formula in the asymmetric unit. Copper(II) cations were found in a pentacoordinated environment with geometrical indices pointing at a very significantly distorted coordination sphere (τ_5_ = 0.61 and 0.46 [32] for Cu1 and Cu2, respectively) (Figure 4). The analysis performed in SHAPE [33] software indicated that Cu1 should rather be described as a trigonal bipyramid (S_TBPY_ = 1.542, S_SPY_ = 3.424), but for Cu2, it did not discriminate unequivocally between the trigonal bipyramid (S_TBPY_ 2.255) and square pyramid (S_SPY_ = 2.236). Both coordination polyhedra shared the common edge due to bridging O22 and O49 oxygen atoms, with Cu1-Cu2 separation (3.090 Å) being even slightly shorter than in **K1**. Hence, both for **K1** and **K2** we can expect significant magnetic interactions. The coordination spheres consisted of two nitrogen atoms and two oxygen atoms from the macrocyclic ring and an apical chloride anion. Cu-N bond lengths ranged from 1.951(6) to 2.077(5) Å, Cu-O bonds were from 1.903(5) to 2.045(5) Å, and Cu-Cl were from 2.302(3) to 2.370(2) Å (Table 2). The naphthyl rings were inclined by 68.1 and 77.5° for C1/C11 and C28/C38 angles, respectively, whereas the angle between C22a and C49a moieties was 30.0°. The mutual orientation of these systems imposed the chirality, and the Flack parameter indicated a pure enantiomer. 

**Figure 6:**
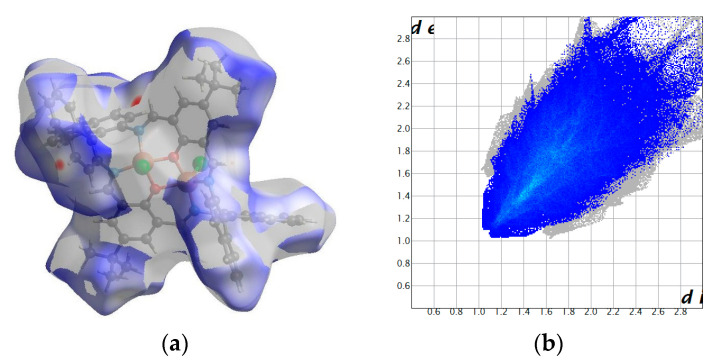

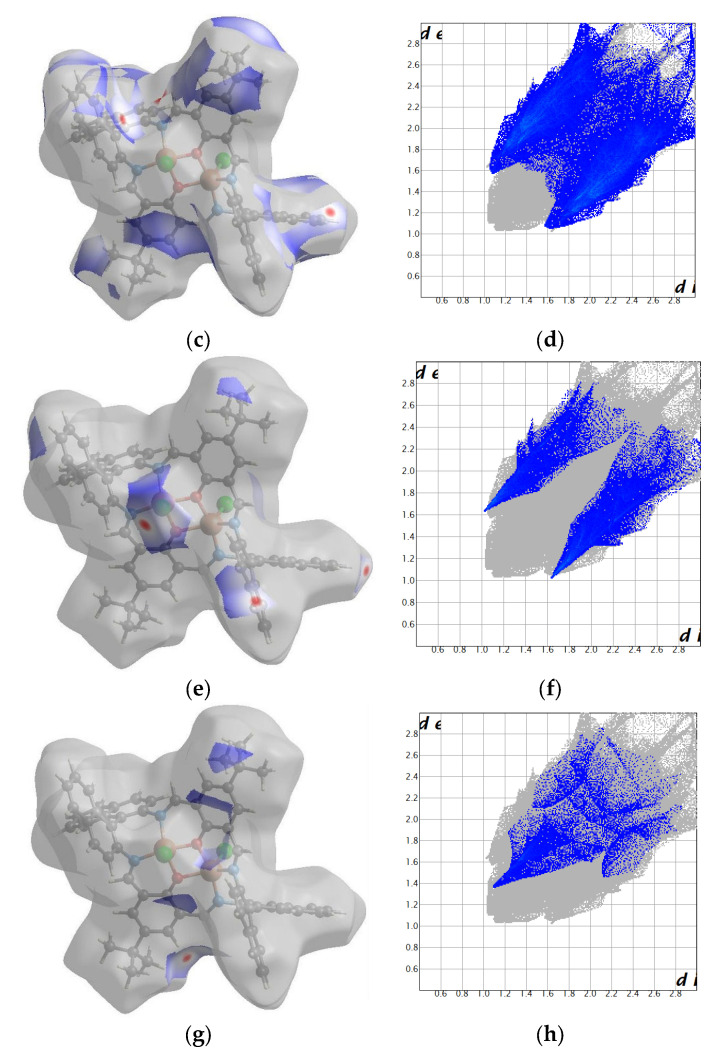
Hirshfeld surfaces and fingerprints of selected interactions created in the crystal network of **K2**: Hirshfeld surface (**a**) and fingerprint (**b**) for H…H (54.2%), Hirshfeld surface (**c**) and fingerprint (**d**) for H…C (28.4%), Hirshfeld surface (**e**) and fingerprint (**f**) for H…Cl (9.0%), and Hirshfeld surface (**g**) and fingerprint (**h**) for H…O (3.6%). In brackets, there is a given surface area included as a percentage of the total surface area.

The packing show channels running along the *b* axis accounted for 461.2 Å^3^ (8.3% of the cell volume) (Figure 5). They were filled with water molecules forming C52-H52…O81[1-x, -1/2+y, 1/2-z] hydrogen bonds. However, there were still some voids (319.1 Å3, 5.8%), indicating that only the O81 water molecule was well defined, whereas there was probably disordered solvent filling those cavities. Hence, fingerprints spanned high di and d_e_ values (Figure 6). The wall of the cavity was formed by two complex molecules. In the network, weak dispersion interactions (H…H and C…H) prevailed due to C-H…π and π…π interactions between strongly inclined aromatic rings (Figure 6). The interaction landscape was completed by Cl…H interactions, with chloride being involved in intermolecular C6-H6…Cl1[-1/2+x, 1/2-y, 1-z] and C32-H32…Cl2[1+x, y, z] hydrogen bonds (red spot on the Hirshfeld surface).

### 2.3. DC Magnetic Measurements, BS DFT Computations, and EPR Spectra

**Figure 7:**
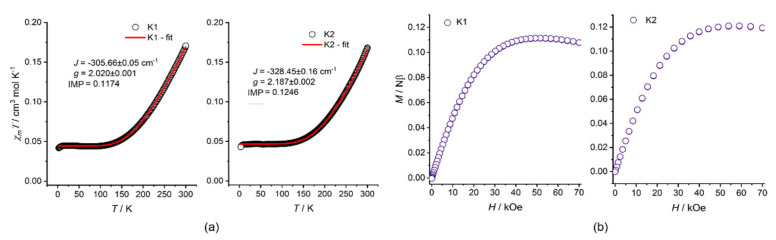
Magnetic properties of **K1** and **K2**: (**a**) *χ_M_T* plots at *H*_dc_ = 5 kOe together with the PHI fits and values of *J*_Cu-Cu_ and *g*_Cu,avg_; (**b**) *M*(*H*) plots at *T* = 1.8 K.

The *χ_M_T* and *M*(*H*) curves for compounds **K1** and **K2** are presented in Figure 7. At *T* = 300 K, the *χ_M_T* product for both compounds was close to 0.17 cm^3^ K mol^–1^, being a value around five times smaller than the 0.867 cm^3^ K mol^–1^ expected for two uncoupled Cu(II) centers assuming *g*_Cu_ = 2.15 within the range typical for 5-coordinative Cu(II) complexes. With the temperature decreasing to around 100 K, the *χ_M_T* products decreased to reach a steady value of 0.044 and 0.047 cm^3^ K mol^–1^ for **K1** and **K2**, respectively. Below 10 K, both curves decreased afresh to reach values slightly above 0.040 cm^3^ K mol^–1^. The shapes of *χ_M_T* were interpreted in terms of very strong magnetic exchange coupling between the Cu(II) centers in the Cu_2_O_2_ coordination cores through the phenoxo-bridges. The low-temperature part of *χ_M_T*, visibly larger than 0, indicated the presence of paramagnetic contribution, which was also confirmed by the *M*(*H*) curves. 

The *χ_M_T* versus *T* curves were fitted in the 1.8–300 K temperature range using the latest PHI software (version 3.1.6, simplex method) [35] and the following Hamiltonian:

$$\hat{H}=-2J_{Cu-Cu}\left({\hat{S}}_{Cu1}\cdot {\hat{S}}_{Cu2}\right)+\mu_{B}Hg_{Cu,avg}\left({\hat{S}}_{Cu1}\cdot {\hat{S}}_{Cu2}\right)$$

where only the magnetic exchange constant between Cu(II) centers, *J*_Cu-Cu_, and the average g_Cu,avg_ factor for both Cu sites were fitted. The additional magnetic impurities, IMP, were included, employing analytical expression for the temperature-dependent magnetic susceptibility, assuming the mononuclear Cu^2+^ complex with g = 2.0. The impurity value represents the fraction of the measured sample **K1** or **K2**, *χ* = (1-IMP) *χ_sample_* + (IMP) *χ_IMP_*. The obtained values are presented in Table 3. 

The obtained *J*_Cu-Cu_ values were in line with the Cu-O-Cu angles, 101.12 and 107.28 deg (av. 104.20 deg) for **K1**, and 99.92 and 107.96 deg (av. 103.94 deg) for **K2** according to archetypal magneto-structural correlation for rhombus Cu_2_O_2_ cores involving hydroxo-, alkoxo-, and phenoxo-bridges [36]. Rather a minor difference of around 25 cm^–1^ between *J*_Cu-Cu_ values of **K1** and **K2** (according to the frames of the above correlation) might conveniently be interpreted in terms of minute differences in the Cu-O distances, Cu-O-Cu angles, and torsion angles within the Cu_2_O_2_ cores (see crystallographic tables). These results were further nicely supported by the broken symmetry and spin flip DFT computational data (Table 3 and Table S1), wherein the *J*(SUP) [37] values close to −337.0 cm^−1^ (**K1**) and −346.5 cm^−1^ (**K2**) estimated according to the spin unprojected (SUP) approach, recommended for the strong coupling regime, provided the best illustration of the J_Cu-Cu_ convergence.

The presence of paramagnetic contribution was confirmed by EPR experiments. The powder spectra recorded for **K1** and **K2** at 77 K, shown in Figure S8, were characteristic for paramagnetic Cu(II) centers. They were successfully simulated with *g_z_* = 2.230, *g_y_* = 2.072, *g_x_* = 2.072, and *A_z_* = 177 × 10^−4^ cm^−1^ for **K1** and *g_z_* = 2.232, *g_y_* = 2.091, *g_x_* = 2.052, and *A_z_* = 173 × 10^−4^ cm^−1^ for **K2**. The parameters observed for the two complexes were similar and indicated the axial N_2_O_2_ coordination environment, which was far less distorted in comparison with dinuclear units, as well as the unpaired electron occupying the molecular orbital with strong contribution from d_x2-y2_ [38,39,40]. Thus, it is suggested that the paramagnetic contribution was due to the fractional incorporation of the monometallic complex of the Cu^II^[Cu^II^_vacancy_]L_2_ or Cu^II^Cl[Cu^II^_vacancy_]L(LH) composition in the crystal, represented by the IMP contribution to the simulation of *χ_M_T*(*T*) data. In comparison to the full model Cu^II^_2_Cl_2_L_2_ complex, such a moiety might contain one empty N_2_O_2_ pocket, denoted as [Cu_vacancy_]. Such a solution relies on the incorporation of around one monometallic complex per eight to nine Cu^II^_2_Cl_2_L_2_ moieties, and is pretty plausible, considering the fact that *topological* shapes of both moieties are sufficiently similar to each other to accommodate the resultant structural defects without the loss of the crystal stability. The above assumption is supported by the fact that optical inspection of the crystals of **K1** and **K2** did not indicate the presence of a separate phase, other than the examined crystals. Moreover, crystal structure solution and refinement allow for the presence of some level of such defects without the loss or even with a minute increase in the solution and refinement quality. The presence of paramagnetic “impurities” was previously observed in dinuclear *S*(^1^/_2_)-S(^1^/_2_) Cu_2_ systems [41,42,43].

The powder EPR spectra of the **K1** and **K2** complexes recorded at 350 K, at which temperature the S = 1 state is highly populated, showed broad, nearly isotropic lines centered at g_eff_ = 2.14 and 2.13, respectively. The peak-to-peak line widths were about 360 G for **K1** and 340 G for **K2** (Figure S9). The broadening of EPR spectra and lack of the resolved *g* tensor anisotropy were caused by spin–spin interactions between relatively close Cu(II) ions arranged in dimeric units in both complexes. Because the spectra did not undergo the resolution of the signals resulting from the resonance transition between the spin states Δ*M_S_* = ±1.0, neither the tensor *g* components nor the zero-field splitting *D* parameter can be determined.

### 2.4. UV–VIS and Fluorescence Spectroscopy

**Figure 8:**
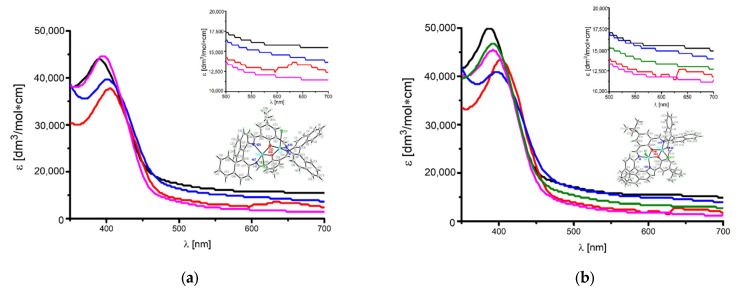
Absorption spectra (**a**) of **K1** and (**b**) **K2** complexes in solvents: chloroform (red), acetone (blue), methanol (black), acetonitrile (green), and DMSO (purple) (3.323 × 10^−6^ mol/dm^3^, RT).

The UV–VIS absorption and fluorescence spectra of the copper(II) complexes were recorded at room temperature in solvents of different polarities: chloroform (ε_r_ = 4.9), acetone (ε_r_ = 20.6), methanol (ε_r_ = 32.7), acetonitrile (ε_r_ = 35.9), and DMSO (ε_r_ = 46.5) [44] (Figure 8, Table S2). Due to the limited solubility of **K1** in acetonitrile, we were unable to record the UV–VIS spectrum in MeCN.

In the UV–VIS spectra of the **K1** and **K2** complexes, bands in the range of 272–290 nm connected with π → π* transitions in the aromatic rings and the bands from the n → π* transitions of the azomethine group between 322 and 324 nm for **K1** and 322 and 328 nm for **K2** were recorded. Bands originating from π → π* transitions in the aromatic rings are typical for this kind of compound and were present in the ligand expected range, and thus we do not show them in Figure 7; only the bands from the charge transfer and d-d transitions are shown.

Band maxima related to the ligand-to-metal charge transfer transition (LMCT) were observed in the range of 388 - 408 nm in a solvent at different polarity: methanol (388 nm), DMSO (398 nm), acetone (402 nm), and chloroform (408 nm) for **K1**, and methanol (388 nm), acetonitrile (392 nm), DMSO (394 nm), acetone (400 nm), and chloroform (404 nm) for **K2** [30]. For both compounds, the bathochromic shift of the LMCT band with a decrease in the solvent polarity (except methanol) was noted (Figure 8, Table S2). This can be connected with the polar character of methanol, which is a protic solvent and causes a different type of interaction with compounds in comparison with the rest of the aprotic solvents [32,45]. Moreover, this can also originate from the distortion of the molecule geometry in the excited state, which implies an increase in the resonance energy and bathochromic shifts.

Additionally, in the absorption spectra, the presence of low-intensity bands at 660 nm for **K1** and 664 nm for **K2** absorption bands from *d-d* transitions [46] only in the less polar solvent chloroform were noted. This results from the square pyramidal or trigonal bipyramid environment of the copper atoms shifted towards the apical chloride anion [23,47]. The same was observed previously for similar copper(II) compounds [41,48].

**Figure 9:**
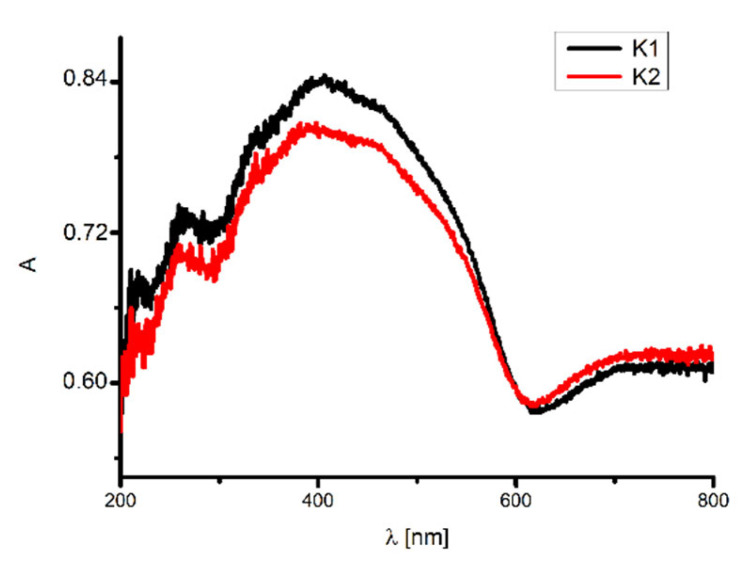
Solid state absorption spectra of black **K1** and red **K2**., A—absorbance.

Furthermore, the UV–VIS spectra of the compounds were also registered at room temperature in the solid state (Figure 9). The spectra show band maxima in the range of 265–266 nm related to π → π* transitions of the aromatic groups and between 385 and 387 nm related to n → π* transitions of the azomethine groups. Moreover, at 468 nm and 461 nm, bands connected with the ligand-to-metal charge transfer LMCT π → d transitions were observed. Above 700 nm for **K1** and 720 nm for **K2**, bands from d-d transitions appeared. The band maxima were shifted towards higher wavelengths by 57–80 nm (LMCT π → d), 63–65 nm (n → π*_C=N_), and 40- 56 nm (d-d) in comparison to the solvents. This shift is connected to the increase in the rigidity of the complexes’ structures in the solid state in comparison to the solution. A red shift of the bands was noted in the spectra of other copper(II) complexes [49].

#### Emission Studies

**Figure 10:**
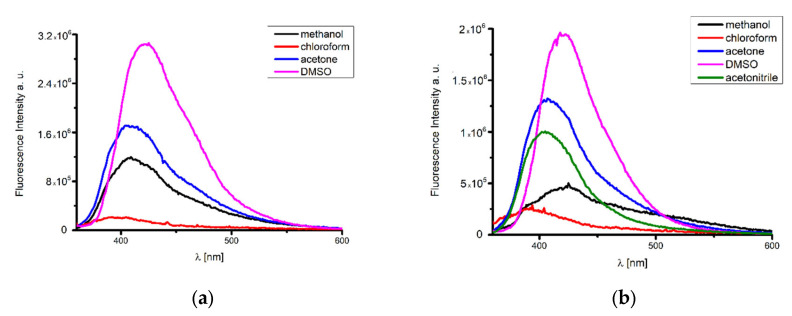
Emission spectra of the (**a**) **K1** and (**b**) **K2** complexes’ solutions; λ_ex_ = 350 nm chloroform, acetone, methanol, acetonitrile, DMSO (3.3 × 10^−6^ mol/dm^3^, RT).

The excitation of **K1** and **K2** in all solvents at 350 nm resulted in blue emission between 398 and 424 nm for **K1** and 392 and 424 nm for **K2** (Figure 10). Emission band maxima of **K1** and **K2** exhibited bathochromic shifts with increasing solvent polarity. Moreover, the highest fluorescence intensity in most polar solvents such as DMSO and methanol was noted (Figure 10, Table S2). A similar situation also occurred in the case of other metal(II) complexes [23]. When the emission spectra registered in a solution and in the solid state were compared, it was possible to infer that the solvent destroyed the π–π interactions, and thus the transition energy was increased in the solution, as was observed previously [50].

### 2.5. Circular Dichroism

**Figure 11:**
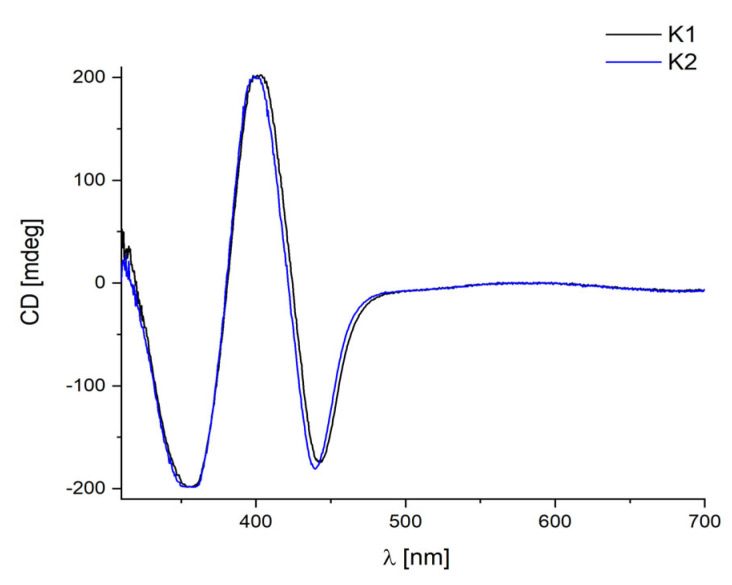
CD spectra of **K1** and **K2** in chloroform 1 × 10^−4^ M.

The chiral character of the studied complexes is reflected in their CD spectra (Figure 11).

The two studied complexes had similar CD spectra with the signals at 314 nm(+24) and 365 nm(−198) for **K1**, and at 311 nm (+18) and 358 nm (−198) for **K2**. The Cotton effect noted above 400 nm at 406(+200) and 444(−174) for **K1***,* and at 403(+198) and 440(−180) for **K2** were connected with n → π*_C=N_ transitions. Conversely, the bands observed at 589 nm for **K1** and 584 nm for **K2** from d-d transitions in Cu ions were a result of a distorted square-pyramidal or trigonal pyramidal geometry [24,51]. The low intense band corresponding to the d–d transitions largely exhibited the same position (≈585 nm) and even a similar intensity for both complexes. This feature can be explained by the existence of an identical {CuN_2_O_2_Cl} chromophore. The replacement of a *tert*-butyl group with a methyl group in the ring did not affect the optical activity of the complexes, which was a consequence of the similarity of the geometry of the studied compounds [52]. Moreover, the signs of the CD bands in the spectra of both complexes **K1** and **K2** were the same because they were derivatives of the same enantiomer of diamine ((*R*)-(+)-1,1′-binaphthalene-2,2′-diamine) [52,53], as was observed for a series of copper(II) complexes with chiral tri- and tetradentate Schiff base ligands derived from 1,1’-binaphthyl-2,2’-diamine [14].

### 2.6. Theoretical Calculations

**Figure 12:**
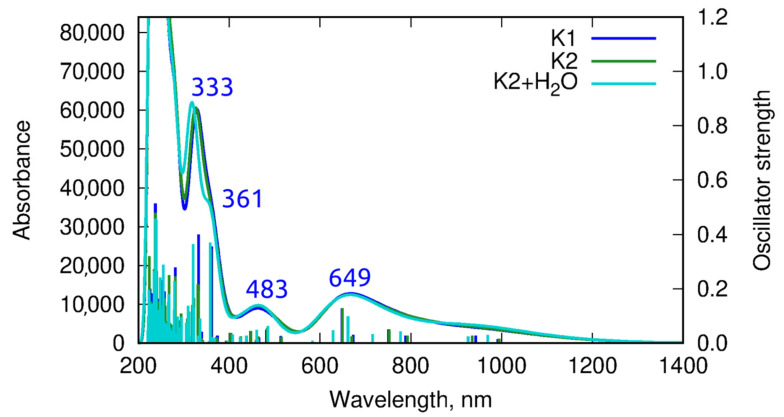
Vertical absorption spectrum for **K1**, **K2,** and **K2** with one water molecule, estimated within the ωB97X-D/def2-SVP/PCM(CHCl_3_) approach (the sticks represent the position and the corresponding oscillator strength for the subsequent excitations, and the curve is the fit to these data).

**Figure 13:**
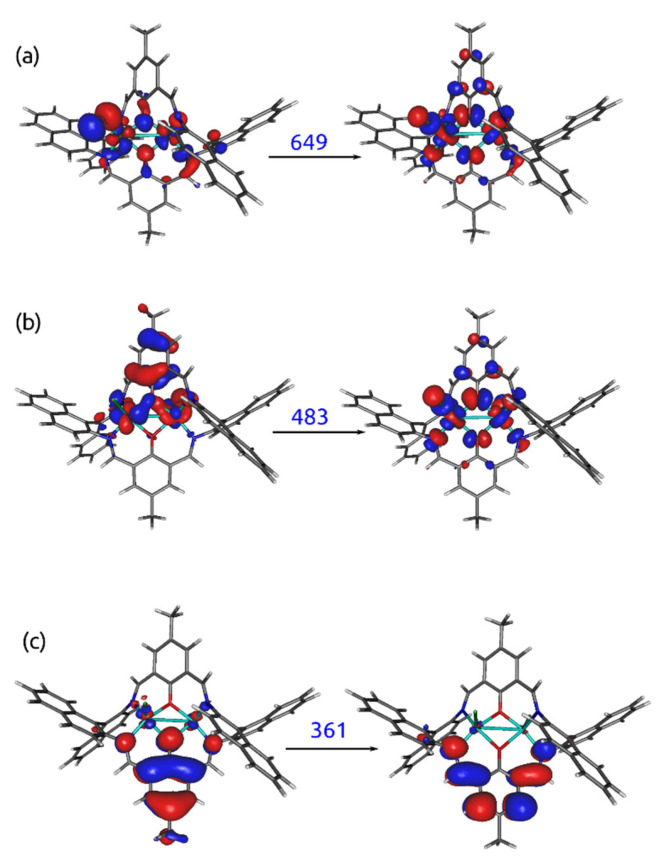
Natural transition orbitals for **K1**, corresponding to the most intensive transitions, estimated within the ωB97X-D/def2-SVP/PCM(CHCl_3_) approach ((**a**) correspond to the d-d* transition, (**b**) to LMCT, and (**c**) to intraligand ππ* transition).

The vertical absorption spectrum estimated within the ωB97X-D/def2-SVP/PCM(CHCl_3_) approach exhibited small intensity signals above 640 nm, corresponding to the d-d* transition forbidden by the Laporte rule (see Figure 12 and the corresponding natural transition orbitals in Figure 13a). The band of about 480 nm involved the transition between the metal and the ligand (the corresponding natural orbitals are presented in Figure 13b). The signals appearing below 400 nm solely arose from the π→π^*^ excitations in the ligand. The shape of the spectrum was only mildly affected by the change of the substituent in the ligand macrocyclic ring from methyl in **K1** to *tert*-butyl in **K2**. The computational results confirmed the assignment provided above on the basis of the experimental measurements in solution.

### 2.7. Thin Materials of Copper(II) Complexes

**Figure 14:**
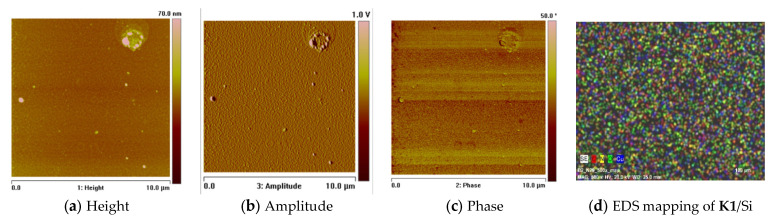
AFM of **K2** material, spin-coating, 3000 rpm, 10 s, chloroform scan size 10 μm, height (thickness) 7 nm, and EDS mapping of **K1**/Si.

**Figure 15:**
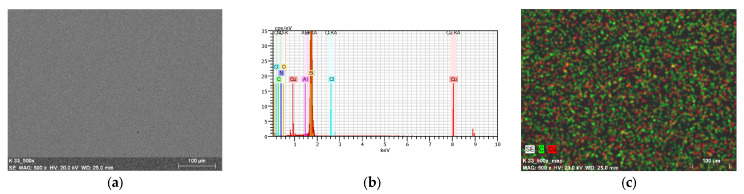
SEM of **K1**/Si thermal deposition. (**a**) **K1**/Si; (**b**) EDS; and (**c**) mapping of **K1**/Si. Magn 500×.

**Figure 16:**
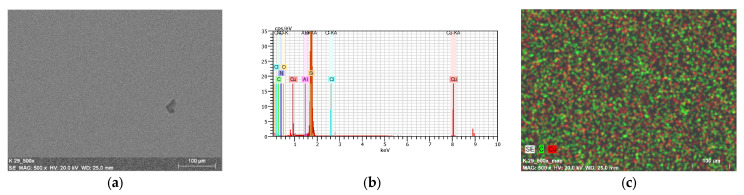
SEM of **K2**/Si thermal deposition. (**a**) **K1**/Si; (**b**) EDS; and (**c**) mapping of **K1**/Si. Magn 500×.

**Figure 17:**
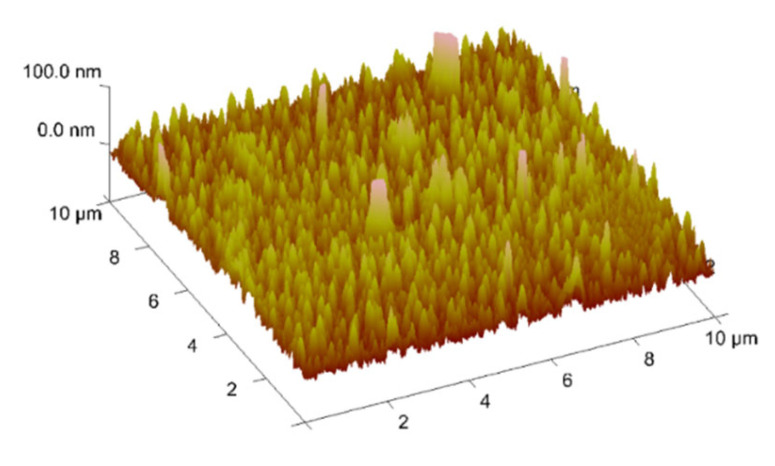

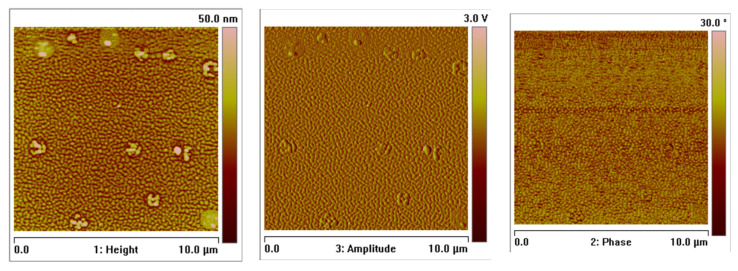
AFM of **K2**/Si, thermal deposition, scan size 10 µm, R_a_ = 4.02 nm, R_q_ = 5.36 nm.

The morphology and roughness of the thin layers were examined by SEM and AFM techniques. To test the chemical composition of materials, the EDS analysis was recorded (Figure 14d, Figure 15, Figure 16, Figures S10 and S11). The optimal parameters of the layers (roughness, parameters, and homogeneity) were obtained in a multi-stage centrifugation spin-coated process using particular parameters: 2500 rpm to 3000 rpm, time of coating 5 or 10 s. Moreover, the thermal vapor deposition method was also used as a second technique to achieve thin materials. The two-dimensional (2D) and three-dimensional (3D) AFM images scanned over a surface area of 1 × 1 µm^2^ are shown in Figure 14, Figure 17 and Figure S12. The values of roughness parameters of materials obtained by thermal deposition were as follows: **K1**/Si R_a_ = 10.7–13.1 nm and R_q_ = 13.2–17.1 nm, and **K2**/Si R_a_ = 3.91–4.96 nm and R_q_ = 4.02–6.38 nm. The roughness of the spin-coated films was similar to that obtained by thermal deposition. However, the spin-coated materials were thinner than those achieved by thermal vapor deposition. A similar situation was noted by us in the case of the layers **L1**/Si or **L2**/Si of the macrocyclic Schiff bases derived from o-phenylenediamine and 2-hydroxy-5-methylisophthalaldehyde **L1** or 2-hydroxy-5-*tert*-butyl-1,3-benzenedicarboxaldehyde **L2** [54].

The values of the roughness parameters for thin materials obtained by both spin-coating and by thermal vapor deposition indicate the achievement of smooth, thin films of copper(II) complexes. Moreover, SEM/EDS analysis showed the presence of carbon, nitrogen, oxygen, and copper in the layer (Figures S10 and S11). SEM/EDS, together with mapping analysis, confirmed the composition of the new materials.

The new films obtained by thermal vapor deposition were also characterized by IR DRIFT (Figure S13). The analysis of the IR DRIFT data showed the presence of the characteristic for the Schiff base peaks between 1653 and 1647 cm^−1^ from stretching frequencies of the azomethine group, and bands from stretching vibrations of aromatic rings ν_C=CAr_ in the region 1568–1485 cm^−1^ were registered. The above-described bands confirmed the presence of the deposited compounds in the obtained materials.

#### Fluorescence Properties of the Materials

**Figure 18:**
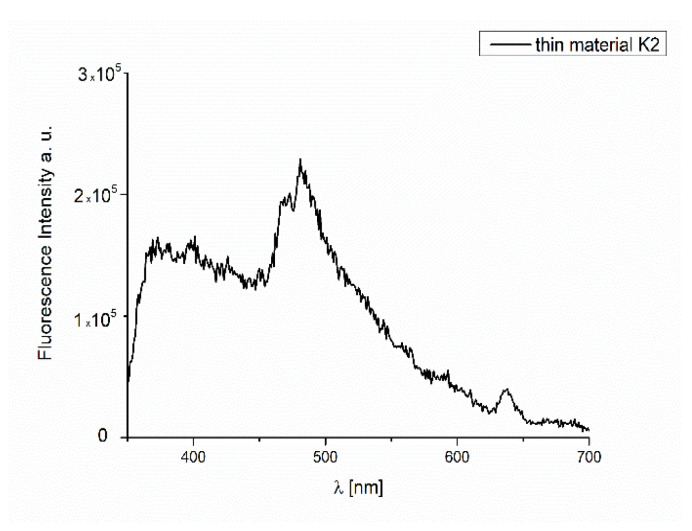
Emission spectra of the **K2** material, λ_ex_ = 320 nm, spin coating.

The fluorescence properties of the thin materials were also studied. The height (thickness) of the spin-coated materials was less than half that of thermally deposited films (70 nm **K1**/Si and 24 nm **K2**/Si), which influenced the emission properties of the composites. The spin-coated **K1**/Si materials did not exhibit fluorescence. Conversely, in the case of **K2**/Si film (Figure 18), the high intensity of the emission bands (λ_ex_ = 320 nm) was observed. 

**Figure 19:**
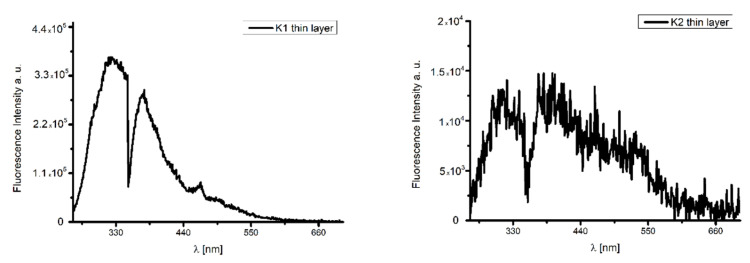
Emission spectra of **K1**/Si and **K2**/Si thermal deposition.

The thin thermally deposited materials showed fluorescence in the range of 318–531 nm for **K1**/Si and 326–472 nm for the **K2** material, λ_ex_ = 250 nm. Furthermore, the emission bands were broad and split into three components (Figure 19, Table S3). The highest intensity of the emission bands for the smooth, thin layer with the equally distributed complexes on the Si surface was noted (Figure 18 and Figure 19). The bathochromic shift of the emission bands of the films in comparison to the solutions was noted. Red shifting of emission maxima was observed for most fluorescent compounds in the solid state, probably due to π–π stacking of the aromatic rings in the molecules [49,54]. An influence of molecular packing in the solid phase on the optical properties can therefore be concluded. This can arise from a different pathway of non-radiative transitions. It can be related to the reduction of the ligand conformational flexibility in the complex. This reduction results from the restraints imposed by the substrate surface. Layers obtained by thermal deposition methods are intended to be used as optical materials.

### 2.8. XAS Analysis

**Figure 20:**
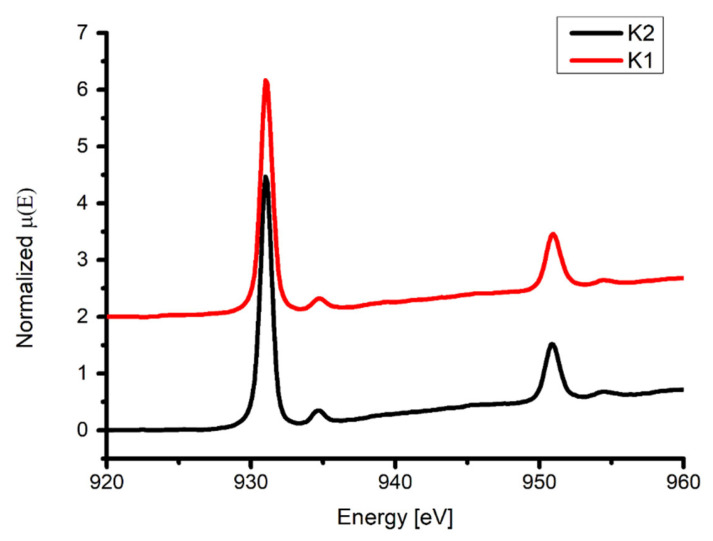
Normalized XANES spectra of copper L_2,3_-edge with peaks corresponding to 2p^6^3d^9^ → 2p^5^3d^10^. The signal for **K1** was shifted by 2 to separate both spectra.

The normalized spectra of **K1** and **K2** were very similar due to the structural similarity of copper coordination spheres in both complexes. Those spectra showed two features for L_3_ and another two for the L_2_ edge (Figure 20, Table 4). In the case of the L_3_ edge, they occurred at 931.0 and 934.8 eV for **K1** and 931.0 and 934.7 eV for **K2**, and the L_2_ features were shifted by around 20 eV towards higher energies. Those values were common for Cu(II) compounds [55,56,57]. According to the theory, the intensity of L_3_ peaks was around twofold stronger than in the case of L_2_, and only one peak related to 2p→3d transitions was expected [58,59,60]. However, additional features are very often observed due to 2p→4s transitions [61,62,63]. Those additional peaks are usually around 25 times weaker due to the smaller cross-section for such X-ray absorption. DeBeer George et al. showed for copper dimers with macrocyclic thiolate ligands and copper ions separated by around 2.9 Å that the L energy pattern depends on the effective nuclear charge the ligand field splitting and also possible metal–metal bonding [64]. Muzioł et al. suggested also that a more complex pattern of the L edge might result from strongly distorted coordination spheres [56]. In the reported structures, we observed such a deformation (see Section 2.2), and the Cu-Cu distance was around 3.1 Å. Hence, we observed much weaker signals following the main features of the L_3_ and L_2_ edges.

## 3. Materials and Methods

2-Hydroxy-5-methylisophthalaldehyde (97%), 2-hydroxy-5-*tert*-butyl-1,3-benzenedicarboxaldehyde (97%), (R)-(+)-1,1′-binaphthyl-2,2′-diamine (99%), and trimethylamine (99.5%) were purchased from Aldrich (Warsaw, Poland), and used without further purification. Copper(II) chloride dihydrate (analytical grade) was supplied by POCh Gliwice, Poland.

### 3.1. Methods and Instrumentation

UV–VIS absorption spectra were recorded in chloroform, acetone, DMSO, acetonitrile, and methanol (3.323 × 10^−6^ M) solutions on a Hitachi spectrophotometer. The fluorescence spectra were recorded on a spectrofluorometer Gildenpλotonics 700 (Dublin, Ireland) in the range 900–200 nm (grating 1, bandpass 5 and 8, integration time 100 ms, chloroform, acetone, DMSO, acetonitrile, and methanol solution of compounds the same as in the case of the UV–VIS studies or silicon slides). The elemental analysis was carried out using a Vario EL III Elemental analyzer. The thermal analysis (TG, DTG, DTA) was performed on an SDT 2960 TA analyzer under air, a heating rate of 10 °C min^−1^, and a heating range of up to 1000 °C and a Jupiter STA 449 F5 thermoanalyzer from Netzsch (Selb, Germany) with an automatic sample feeder coupled to a Vertex 70V FT-IR spectrometer from Bruker Optik (Ettlingen, Germany). After combustion, the residue of the sample was analyzed by an XRD analysis performed with a Philips X’Pert equipped with an X’Celerator Scientific detector. The IR spectra were recorded on the Bruker instrument using the ATR technique in the range of 70–4000 cm^−1^. Circular dichroism spectra were recorded with a Jasco J-815 spectropolarimeter (Jasco Inc.) in the range of 310–700 nm wavelengths. The solution of **K1** and **K2** complexes (≈1 × 10^−4^ M) was prepared by dissolving it in a CHCl_3_ solution.

#### 3.1.1. Spin Coating

Layers of the complexes were deposited on Si(111) wafers (10 nm × 10 mm) that were ≈500 nm thick using the spin coating technique. Precursors were dissolved in chloroform and deposited on Si using a spin coater (Laurell 650 SZ). The spin speed varied from 2500 to 3000 rpm, and the coating time was 5 or 10 s. 

#### 3.1.2. Thermal Vapor Deposition

The thin layer of **K1** and **K2** was deposited on n-type silicon substrate. The orientation of the silicon substrate was (100) with electrical resistivity (ρ) equal to 6.2 × 10^−3^ Ω cm. The silicon wafer was first degreased in acetone, ethanol, and finally in deionized water using an ultrasonic bath. On the front side (polished side) of the silicon wafer, a **K1** and **K2** layers of 24–70 nm thickness were deposited in a vacuum (p = 2 × 10^−4^ Pa) by a thermal evaporation method, with an evaporation rate of 0.2 nm/s, without heating of the substrate.

The morphology and composition of the obtained films were analyzed with a scanning electron microscope (SEM; LEO Electron Microscopy Ltd, Cambridge, UK), the 21430 VP model equipped with secondary electrons (SE) detectors, and an energy-dispersive X-ray spectrometer (EDX) Quantax with an XFlash 4010 detector (Bruker AXS microanalysis GmbH, Berlin, Germany). The atomic force microscopy (AFM) images were performed in the tapping mode with a Multi Mode Nano Scope IIIa (Veeco Digital Instrument, SB, US) microscope. The structure of the produced layers was estimated using diffuse reflectance infrared Fourier transform spectroscopy (DRIFT, Spectrum 2000, PerkinElmer Inc., Waltham, MA, USA). The absorption spectra of the solid samples **K1** and **K2** were recorded on the Jasco V-750 spectrophotometer in the 200–800 nm (bandwidth 1.0 nm, data interval 0.2 nm).

### 3.2. Crystal Structure Determination

The diffraction data of the studied compounds were collected for the single crystal at 100 K using an XtaLAB Synergy Dualflex equipped with a HyPix detector and CuK_α_ source (λ = 1.54184 Å) for **K1** and on BL14.2 beamline (Helmholtz Zentrum Berlin, Bessy II) operating at λ = 0.7999 Å for **K2**. For **K1**, the full process of the data reduction was performed in CrysAlis Pro [65], whereas for **K2**, the data reduction and space group determination were performed with *xdsapp* [66,67], and then CrysAlis Pro was used for final data reduction. For **K2,** the absorption correction was introduced by the diffabs method implemented into WinGx [68]. The structure was solved by the direct methods and refined with the full-matrix least-squares procedure on F^2^ (SHELXL-2018/1) [69]. All heavy atoms were refined with anisotropic displacement parameters. Hydrogen atoms were located at calculated positions with thermal displacement parameters fixed to a value of 20% or 50% higher than those of the corresponding carbon atoms. It should be noted that for **K1,** the bypass procedure implemented into Olex2 [34] was applied due to poorly defined density in the solvent region of the porous complex. It resulted in significant amelioration of the final model. However, the whole interaction model suffered from missing solvent–complex and solvent–solvent interactions. In **K2**, the O81 water molecule was refined with partial occupancy (0.5), and the O21 molecule was located very close to the inversion center. In the final model of **K2**, there were missing hydrogen atoms from the O81 water molecule. The stable refinement was achieved with ISOR and DELU restraints for the positionally disordered C(61)H_3_ methyl group in **K1** and for (ISOR) for C62, C64, and C74 atoms from *tert*-butyl groups of **K2**. All figures were prepared in DIAMOND [70] and ORTEP-3 [71]. The results of the data collection and refinement are summarized in Table 5.

### 3.3. SQUID Measurements

Magnetic measurements were investigated using a Quantum Design MPMS 3 SQUID magnetometer. For the measurements, powdered compounds **K1** and **K2** were sealed in two foil bags. The dc magnetic susceptibilities were measured in the 300–1.8K temperature range with an applied field of 5 kOe. The measurements were carried out in sweep mode with a scan rate of 2 K min^–1^. The magnetization curves were measured in the 0–70 kOe range with dc field stabilization. Magnetic data were corrected for the diamagnetic contribution of the plastic bags and sample by empirical and Pascal’s constants, respectively [72]. Moreover, the TIP contribution of 5.9 × 10^−5^ cm^3^ mol^−1^ for Cu(II) metal ion was applied.

### 3.4. EPR Measurements

EPR experiments were carried out for powder samples using a Bruker Elexsys E500 spectrometer operating at ∼9.6 GHz (X-band) frequency. The spectrometer was equipped with an NMR teslameter and a frequency counter. The temperature was controlled by using a finger-Dewar for measurements at 77 K and a Bruker ER 4131VT variable temperature accessory for 350 K. We set the amplitude and frequency of the modulating field to 10 G and 100 kHz, respectively, and we set the microwave power to 20 mW. The EPR spectra were simulated using EasySpin 5.2.35 [73,74].

### 3.5. Theoretical Calculations

The full geometry optimization of **K1** and **K2** was performed within the ωB97X-D/def2-SVP approach in a vacuum for magnetic coupling analysis and in the PCM model of chloroform for photophysical properties, starting from the crystal structure. The character of the stationary points was confirmed with the harmonic vibration analysis. Vertical absorption was investigated within the same approach, and ECD spectra were depicted for the analyzed enantiomers. The corresponding natural transition orbitals were examined in order to determine the character of the most intensive transitions. All these calculations were carried out with the Gaussian16 program [75]. The magnetic coupling parameters of the complexes were estimated within the broken symmetry approach and spin-flip formalism in B3LYP functional, according to the recommendations of Neese et al. [76] for the gas phase optimized geometry of both complexes. Moreover, the moderate-size def2-SVP basis set was applied for these calculations for the light atoms, as it has been proven to provide a good cost-to-quality balance for large systems containing copper atoms, and the basis set for copper was extended to the def2-QZVPP one [76]. Moreover, the spin-flip formalism was also employed with the same functional and basis set, for comparison. The magnetic coupling constants J were estimated according to spin-unprojected (SUP) scheme proposed by Ruiz for the strong coupling regime [37,77]. The corresponding calculations are based on the total spin-coupling Hamiltonian of the form -2J(Cu_1_-Cu_2_)S_Cu1_S_Cu2_, where J(Cu_1_-Cu_2_) is the exchange coupling constant between the two copper atoms with total spins S_Cu1_ and S_Cu2_, individually. Additionally, the spin-projected scheme and approximated spin-projected scheme were also applied for comparison, and the corresponding data are presented in the Supporting Information. All the magnetic coupling constant calculations were carried out with the Orca program package [78]. 

### 3.6. XAS

X-ray absorption spectra were recorded at the National Synchrotron Radiation Centre SOLARIS at the bending magnet PIRX beamline equipped with a collimated Plane Grating Monochromator for a copper L2,3-edge (910–1040 eV). The sample was finely ground and attached to double-sided adhesive conductive graphite tape. The measurements were performed with the step size of 0.25 eV for the pre-edge region, 0.15 eV for the edge regions, and 0.5 eV for the high energy part. The data sets were collected at room temperature in an ultra-high vacuum (UHV) using total electron yield mode (TEY). The data were processed using the ATHENA program from the Demeter package [79]. 

## 4. Experimental

### Synthesis of Complexes

**K1**

A total of 0.5 mmol of 2-hydroxy-5-methylisophthalaldehyde, 0.5 mmol of (*R*)-(+)-1,1′-binaphthalene-2,2′-diamine, 0.5 mmol of copper(II) chloride dihydrate, and excess of triethylamine were dissolved in 80 cm^3^ of methanol. The synthesis was carried out under reflux for 1 hour. The product was dried under air, and single brown crystals were received. (yield: 94%). m. p. > 350 °C. C_58_H_38_Cl_2_Cu_2_N_4_O_2_x4 H_2_O (calc./found %): C 63.73/64.08, N 5.12/5.16.

Selected FT-IR (data reflectance, crystal) (cm^−1^), 3053, 3039, 2951, 2921 ν_C-HAr_, 1616 ν_C=N_, 1540, 1502, 1467ν_C=CAr_, 1319 ν_Ph-O_ (Figure S1).

**K2**

A total of 0.5 mmol of 4-*tert*-butyl-2,6-diformylphenol, 0.5 mmol of (R)-(+)-1,1′-binaphthalene-2,2′-diamine, 0.5 mmol of copper(II) chloride dihydrate, and excess of triethylamine were dissolved in 80 cm^3^ of methanol. The synthesis was carried out under reflux for 1 hour. The product was dried under air, and single brown crystals were crystalized in slow evaporation using a mixture of acetonitrile/chloroform and determined by crystal analysis. (yield: 90%). m. p. > 350 °C. C_64_H_52_Cl_2_Cu_2_N_4_O_3_ (calc./found %): C 68.44/68.69, N 4.99/5.12.

Selected FT-IR (data reflectance, crystal) (cm^−1^), 3050, 3040, 2952, 2865 ν_C-HAr_, 1675 ν_C=N_, 1581, 1502, 1466ν_C=CAr_, 1322 ν_Ph-O_ (Figure S2).

## 5. Conclusions

The research presented herein describes the synthesis of two new macrocyclic dinuclear copper(II) complexes, with N_4_O_2_ donor atoms. The Cu(II) complexes **K1** and **K2** were prepared by the template reaction of (*R*)-(+)-1,1′-binaphthalene-2,2′-diamine and 2-hydroxy-5-methyl-1,3-benzenedicarboxaldehyde **K1**, or 4-*tert*-butyl-2,6-diformylphenol **K2** with copper(II) chloride dihydrate. The X-ray data show that copper(II) cations are in a heavily distorted pentacoordinated environment. The coordination spheres consist of two nitrogen atoms and two oxygen atoms from the macrocyclic ring and apical chloride anion. In the case of **K1,** the voids accounting for 1231.5 Å^3^ (22.7% of the volume cell) were noted, whereas for **K2,** channels running along the *b* axis accounted for 461.2 Å3 (8.3% of the cell volume) filled with water molecules forming C52-H52…O81[1-x, -1/2+y, 1/2-z] hydrogen bonds existed. The magnetic studies showed very strong antiferromagnetic Cu^II^-C^II^ exchange interactions represented by *J*_Cu-Cu_ = −305.66 ± 0.05 cm^−1^ for **K1** and *J*_Cu-Cu_ = −328.45 ± 0.16 cm^−1^ for **K2,** which were nicely correlated with structural data and confirmed by the broken symmetry DFT calculations. The EPR spectra of the complexes **K1** and **K2** showed broad, nearly isotropic lines centered at g_eff_ = 2.14 and 2.13, respectively. The broadening of EPR spectra and lack of the resolved *g* tensor anisotropy were caused by spin–spin interactions between relatively close Cu(II) ions arranged in dimeric units in both complexes. The compliance between experimental and theoretical results validated the developed calculation method, which will be used to design new binuclear copper(II) complexes. The obtained complexes exhibited emission in the various polarity solvents and in the solid state. The blue emission between 398 and 424 nm for **K1** and 392 and 424 nm for **K2** in a solvent at different polarities was observed. The bathochromic shift with increasing solvent polarity for **K2** was also observed. The highly ordered materials were obtained by spin-coating and thermal vapor methods, exhibiting fluorescence in the range of 318–531 nm for **K1**/Si and 326–472 nm for the **K2**/Si material, λ_ex_ = 250 nm (thermally deposited films). The emission bands were broad and split into three components. The bathochromic shift of the fluorescence bands of films in comparison to that of the solution was noted. This can result from molecular packing in the solid state being different to that in the solution. The fluorescence emission of the layers makes these films potentially suitable for application in light-emitting devices. 

## Figures and Tables

**Table 1:** Selected bond length (Å) and valence angles (°) for the complex **K1**.

(K1)			
Cu(1)-N(1)	1.917(11)	Cu(2)-O(49)	2.053(8)
Cu(1)-O(22)	1.944(8)	Cu(2)-N(28)	2.069(9)
Cu(1)-O(49)	1.974(6)	Cu(2)-Cl(2)	2.363(2)
Cu(1)-N(20)	2.034(10)		
Cu(1)-Cl(1)	2.495(3)		
Cu(2)-O(22)	1.923(7)		
Cu(2)-N(47)	1.954(9)		
	Valence angles (°)		
N(1)-Cu(1)-O(22)	168.5(3)	O(49)-Cu(1)-Cl(1)	121.48(18)
N(1)-Cu(1)-O(49)	92.4(3)	N(20)-Cu(1)-Cl(1)	95.0(3)
O(22)-Cu(1)-O(49)	76.2(3)	O(22)-Cu(2)-N(47)	165.5(3)
N(1)-Cu(1)-N(20)	101.8(4)	O(22)-Cu(2)-O(49)	74.8(3)
O(22)-Cu(1)-N(20)	87.5(4)	N(47)-Cu(2)-O(49)	91.0(3)
O(49)-Cu(1)-N(20)	140.3(3)	O(22)-Cu(2)-N(28)	89.3(3)
N(1)-Cu(1)-Cl(1)	92.7(2)	N(47)-Cu(2)-N(28)	98.8(3)
O(22)-Cu(1)-Cl(1)	93.2(2)	O(49)-Cu(2)-N(28)	134.7(3)

**Table 2:** Selected bond length (Å) and valence angles (°) for the complex **K2**.

K2			
Cu(1)-O(22)	1.920(5)	Cu(2)-O(49)	1.989(5)
Cu(1)-N(1)	1.953(6)	Cu(2)-N(28)	2.061(6)
Cu(1)-O(49)	2.047(5)	Cu(2)-Cl(2)	2.370(3)
Cu(1)-N(20)	2.078(5)		
Cu(1)-Cl(1)	2.302(3)		
Cu(2)-O(22)	1.900(5)		
Cu(2)-N(47)	1.954(7)		
	Valence angles (°)		
O(22)-Cu(1)-N(1)	165.2(2)	O(49)-Cu(1)-Cl(1)	115.4(2)
O(22)-Cu(1)-O(49)	75.1(2)	N(20)-Cu(1)-Cl(1)	113.60(19)
N(1)-Cu(1)-O(49)	90.4(2)	O(22)-Cu(2)-N(47)	165.0(3)
O(22)-Cu(1)-N(20)	87.7(2)	O(22)-Cu(2)-O(49)	77.0(2)
N(1)-Cu(1)-N(20)	99.3(2)	N(47)-Cu(2)-O(49)	89.5(3)
O(49)-Cu(1)-N(20)	128.7(3)	O(22)-Cu(2)-N(28)	87.4(2)
O(22)-Cu(1)-Cl(1)	93.74(19)	N(47)-Cu(2)-N(28)	98.5(3)
N(1)-Cu(1)-Cl(1)	95.4(2)	O(49)-Cu(2)-N(28)	137.4(3)

**Table 3:** Fitted values of *J*_Cu-Cu_, g_Cu,avg_, and IMP contribution. The *J*(SUP) ^a^ was extracted from the broken symmetry and spin-flip DFT results (for details, see SI section).

Compound	K1	K2
*J*_Cu-Cu_/cm^−1^	−305.66 ± 0.05	−328.45 ± 0.16
*J*(SUP) ^a^	−337.0	−346.5
*g*_Cu,avg_	2.020 ± 0.001	2.187 ± 0.002
IMP ^b^/%	11.74	12.46
Residual ^c^	6.35 ± 10^−4^	3.79 ± 10^−4^

^a^ Spin-unprojected approximation. ^b^ Paramagnetic contribution represented by the option of “impurities” in PHI software. ^c^ The value of the fit quality test function embedded in PHI software.

**Table 4:** Summary of spectral features of the Cu absorption at the L-edges (peak energies and intensities).

Compound	Energy of Maximum L_3_-Edge (eV) (L_3_-Edge Intensity)	Energy of Maximum L_2_-Edge (eV) (L_2_-Edge Intensity)
**K1**	931.0 (4.17) 934.8 (0.33)	951.0 (1.46) 954.5 (0.65)
**K2**	931.0 (4.47) 934.7 (0.35)	950.9 (1.51) 954.5 (0.68)

**Table 5:** Crystal data and structure refinement for **K1** and **K2**.

Identification Code	K1	K2
Empirical formula	C_58_ H_38_ Cl_2_ Cu_2_ N_4_ O_2_	C_64_ H_52_ Cl_2_ Cu_2_ N_4_ O_3_
Formula weight	1020.90	1123.07
Temperature (K)	100(2)	100(2)
Wavelength (Å)	1.54184	0.7999
Crystal system, space group	Orthorhombic, P2_1_2_1_2	Orthorhombic, P2_1_2_1_2_1_
Unit cell dimensions (Å) and (°)	a = 10.6153(3) α = 90 b = 22.3313(15) β = 90 c = 22.9053(11) γ = 90	a = 10.922(2) α = 90 b = 19.448(4) β = 90 c = 26.041(5) γ = 90
Volume (Å^3^)	5429.8(5)	5531.4(19)
Z, Calculated density (Mg⋅m^–3^)	4, 1.249	4, 1.349
Absorption coefficient (mm^–1^)	2.207	1.262
F(000)	2088	2302
Crystal size (mm^3^)	0.150 × 0.050 × 0.030	0.170 × 0.070 × 0.070
Theta range for data collection (°)	2.764 to 68.243	1.471 to 28.430
Limiting indices	−10 ≤ h ≤ 12 −26 ≤ k ≤ 26 −27 ≤ l ≤ 27	−12 ≤ h ≤ 12 −23 ≤ k ≤ 23 −30 ≤ l ≤ 30
Reflections collected/unique	36173/9932 (R(int) = 0.0997)	61117/9714 (R(int) = 0.0538)
Completeness to theta = 29.732° (%)	99.8	99.5
Max. and min. transmission	1.000 and 0.672	0.8864 and 0.2903
Refinement method	Full-matrix least-squares on F^2^	Full-matrix least-squares on F^2^
Data/restraints/parameters	9932/13/617	9714/18/679
Goodness-of-fit on F^2^	0.979	1.068
Final R indices (I > 2sigma(I))	R1 = 0.0795, wR2 = 0.1930	R1 = 0.0581, wR2 = 0.1449
R indices (all data)	R1 = 0.1161, wR2 = 0.2166	R1 = 0.0664, wR2 = 0.1501
Largest diff. peak and hole (eÅ^–3^)	1.420 and −0.434	0.602 and −0.481

CCDC 2233358 and 2233362 contain the supplementary crystallographic data for **K1** and **K2**. These data can be obtained free of charge from The Cambridge Crystallographic Data Centre via www.ccdc.cam.ac.uk/data_request/cif (accessed on 29 December 2022).

## Data Availability

The data presented in this study are available in Supplementary Materials.

## Supplementary Materials

The following supporting information can be downloaded at: https://www.mdpi.com/article/10.3390/ijms24033017/s1.

## References

[1] Liu X., Hamon J.R. (2019). Recent developments in penta-, hexa- and heptadentate Schiff base ligands and their metal complexes. Coord. Chem. Rev..

[2] Gregoliński J., Ślepokura K., Kłak J., Witwicki M. (2022). Multinuclear Ni(II) and Cu(II) complexes of a *meso* 6 + 6 macrocyclic amine derived from *trans*-1,2-diaminocyclopentane and 2,6-diformylpyridine. Dalton Trans..

[3] Vigato P., Tamburini S., Bertolo L. (2007). The development of compartmental macrocyclic Schiff bases and related polyamine derivatives. Coord. Chem. Rev..

[4] Chang F.-F., Zhang K., Huang W. (2018). Schiff-base macrocyclic Zn(II) complexes based upon flexible pendant-armed extended dialdehydes. Dalton Trans..

[5] Löffler M., Gregoliñski J., Korabik M., Lis T., Lisowski J. (2016). Multinuclear Ni(II), Cu(II) and Zn(II) complexes of chiral macrocyclic nonaazamine. Dalton Trans..

[6] Das M., Mukherjee S., Koley B., Choudhuri I., Bhattacharyya N., Roy P., Samanta B.C., Baraif M., Maity T. (2020). Developing novel zinc(II) and copper(II) Schiff base complexes: Combined experimental and theoretical investigation on their DNA/protein binding efficacy and anticancer activity. New J. Chem..

[7] Zhang J., Xu L., Wong W.-Y. (2018). Energy materials based on metal Schiff base complexes. Coord. Chem. Rev..

[8] Janczak J., Prochowicz D., Lewiński J., Fairen-Jimenez D., Bereta T., Lisowski J. (2016). Trinuclear cage-like ZnII macrocyclic complexes: Enantiomeric recognition and gas adsorption properties. Eur. J. Chem..

[9] Zhang X., Yin J., Yoon J. (2014). Recent Advances in Development of Chiral Fluorescent and Colorimetric Sensors. Chem. Rev..

[10] Li Z.-B., Lin J., Sabat M., Hyacinth M., Pu L. (2007). Enantioselective Fluorescent Recognition of Chiral Acids by Cyclohexane-1,2-diamine-Based Bisbinaphthyl Molecules. J. Org. Chem..

[11] Liu X., Manzur C., Novoa N., Celedón S., Carrillo D., Hamon J.R. (2018). Multidentate unsymmetrically-substituted Schiff bases and their metal complexes: Synthesis, functional materials properties, and applications to catalysis. Coord. Chem. Rev..

[12] Padnya P., Shibaeva K., Arsenyev M., Baryshnikova S., Terenteva O., Shiabiev I., Khannanov A., Boldyrev A., Gerasimov A., Grishaev D. (2021). Catechol-Containing Schiff Bases on Thiacalixarene: Synthesis, Copper (II) Recognition, and Formation of Organic-Inorganic Copper-Based Materials. Molecules.

[13] Che C.-M., Huang J.-S. (2003). Metal complexes of chiral binaphthyl Schiff-base ligands and their application in stereoselective organic transformations. Coord. Chem. Rev..

[14] Shockravi A., Javadi A., Abouzari-Lotf E. (2013). Binaphthyl-based macromolecules: A review. RSC Adv..

[15] Hajari S., Keypour H., Rezaei M.T., Farida S.H.M., Gable R.W. (2022). New 15-membered macrocyclic Schiff base ligand; synthesis some Cd(II), Mn(II) and Zn(II) complexes, crystal structure, cytotoxicity, antibacterial and antioxidant activity. J. Mol. Struct..

[16] El-Gammal O.A., Mohamed F.S., Rezk G.N., El-Bindary A.A. (2021). Synthesis, characterization, catalytic, DNA binding and antibacterial activities of Co(II), Ni(II) and Cu(II) complexes with new Schiff base ligand. J. Mol. Liq..

[17] Lee E., Lee S.Y., Lindoy L.F., Lee S.S. (2013). Metallacycles derived from metal complexes of exo-coordinated macrocyclic ligands. Coord. Chem. Rev..

[18] Tokunaga H., Kazama K., Tsuboi M., Miyasaka M. (2021). A novel Schiff base macrocycle based on 1,10 -binaphthyl for fluorescence recognition. Luminescence.

[19] Korupoju S.R., Mangayarkarasi N., Ameerunisha S., Valente E.J., Zacharias P.S. (2000). Formation of dinuclear macrocyclic and mononuclear acyclic complexes of a new trinucleating hexaaza triphenolic Schiffbase macrocycle: Structure and NLO properties. J. Chem. Soc. Dalton Trans..

[20] Telfer S.G., Sato T., Harada T., Kuroda R., Lefebvre J., Leznoff D.B. (2004). Mono- and Dinuclear Complexes of Chiral Tri- and Tetradentate Schiff-Base Ligands Derived from 1,1’-Binaphthyl-2,2’-diamine. Inorg. Chem..

[21] Telfer S.G., Kuroda R. (2003). 1,1′-Binaphthyl-2,2′-diol and 2,2′-diamino-1,1′-binaphthyl: Versatile frameworks for chiral ligands in coordination and metallosupramolecular chemistry. Coord. Chem. Rev..

[22] Chinnaraja E., Arunachalam R., Suresh E., Sen S.K., Natarajan R., Subramanian P.S. (2019). Binuclear Double-Stranded Helicates and Their Catalytic Applications in Desymmetrization of Mesodiols. Inorg. Chem..

[23] Chinnaraja E., Arunachalam R., Samanta K., Natarajan R., Subramaniana P.S. (2020). Enantioselective Michael Addition Reaction Catalysed by Enantiopure Binuclear Nickel(II) Close-Ended Helicates. Adv. Synth. Catal..

[24] Chinnaraja E., Arunachalam R., Pillai R.S., Peuronen A., Rissanen K., Subramanian P.S. (2020). One-pot synthesis of [2+2]-helicate-like macrocycle and 2+4-μ4-oxo tetranuclear open frame complexes: Chiroptical properties and asymmetric oxidative coupling of 2-naphthols. Appl. Organomet. Chem..

[25] Castellano M., Ruiz-García R., Cano J., Ferrando-Soria J., Pardo E., Fortea-Pérez F.R., Stiriba S.-E., Julve M., Lloret F. (2015). Dicopper(II) Metallacyclophanes as Multifunctional Magnetic Devices: A Joint Experimental and Computational Study. Acc. Chem. Res..

[26] Ferrando-Soria J., Vallejo J., Castellano M., Martínez-Lillo J., Pardo E., Cano J., Castro I., Lloret F., Ruiz-García R., Julve M. (2017). Molecular magnetism, *quo vadis?* A historical perspective from a coordination chemist viewpoint. Coord. Chem. Rev..

[27] Žilić D., Rakvin B., Milić D., Pajić D., Đilović I., Camettid M., Džolić Z. (2014). Crystal structures and magnetic properties of a set of dihalo-bridged oxalamidato copper(II) dimers. Dalton Trans..

[28] Brown S.J., Tao X., Wark T.A., Stephan D.W., Mascharak P.K. (1988). Synthetic analog approach to metallobleomycins. 4. New halobridged dimeric and polymeric (infinite zigzag chain) complexes of copper(II) with peptide ligands related to bleomycins. Inorg. Chem..

[29] Sena N., Butcher J.R., Jasinski J.P., Gupta S.K. (2021). A novel single-pot synthesis of dinuclear and mononuclear copper(II) complexes with sterically demanding Schiff bases: Structural, spectral, magnetic, electrochemical, DNA binding and theoretical investigation. J. Mol. Struct..

[30] Chattopadhyay T., Banu K.S., Banerjee A., Ribas J., Majee A., Nethaji M., Das D. (2007). A novel single pot synthesis of binuclear copper(II) complexes of macrocyclic and macrocyclic compartmental ligands: Structures and magnetic properties. J. Mol. Struct..

[31] Thompson K., Mandal S.K., Tandon S.S., Bridson J.N., Park M.K. (1996). Magnetostructural Correlations in Bis(µ2-phenoxide)-Bridged Macrocyclic Dinuclear Copper(II) Complexes. Influence of Electron-Withdrawing Substituents on Exchange Coupling Laurence. Inorg. Chem..

[32] Addison A.W., Rao T.N., Reedijk J., van Rijn J., Verschoor G.C. (1984). Synthesis, structure, and spectroscopic properties of copper(II) compounds containing nitrogen–sulphur donor ligands; the crystal and molecular structure of aqua[1,7-bis(N-methylbenzimidazol-2′-yl)-2,6-dithiaheptane]copper(II) perchlorate. J. Chem. Soc. Dalton Trans..

[33] Llunel M., Casanova D., Cirera J., Bofill J.M., Alemany P., Alvarez S., Pinsky M., Avnir D. (2013). SHAPE, Version 2.1.

[34] Dolomanov O.V., Bourhis L.J., Gildea R.J., Howard J.A.K., Puschmann H. (2009). OLEX2: A complete structure solution, refinement and analysis program. J. Appl. Cryst..

[35] Chilton N.F., Anderson R.P., Turner L.D., Soncini A., Murray K.S.J. (2013). PHI: A powerful new program for the analysis of anisotropic monomeric and exchange-coupled polynuclear d- and f-block complexes. Comput. Chem..

[36] Kahn O. (1993). Molecular Magnetism.

[37] Ruiz E., Cano J., Alvarez S., Alemany P. (1999). Broken symmetry approach to calculation of exchange coupling constants for homobinuclear and heterobinuclear transition metal complexes. J. Comput Chem..

[38] Maślewski P., Wyrzykowski D., Witwicki M., Dołęga A. (2018). Histaminol and Its Complexes with Copper(II)—Studies in Solid State and Solution. Eur. J. Inorg. Chem..

[39] Mielcarek A., Bieńko A., Saramak P., Jezierska J., Dołęga A. (2019). A Cu/Zn heterometallic complex with solvent-binding cavity, catalytic activity for the oxidation of 1-phenylethanol and unusual magnetic properties. Dalton Trans..

[40] Peisach J., Blumberg W.E. (1974). Structural implications derived from the analysis of electron paramagnetic resonance spectra of natural and artificial copper proteins. Arch. Biochem. Biophys..

[41] Barwiolek M., Kaczmarek-Kędziera A., Muziol T.M., Jankowska D., Jezierska J., Bieńko A. (2020). Dinuclear Copper(II) Complexes with Schiff Bases Derived from 2-Hydroxy-5-Methylisophthalaldehyde and Histamine or 2-(2-Aminoethyl)pyridine and Their Application as Magnetic and Fluorescent Materials in Thin Film Deposition. Int. J. Mol. Sci..

[42] Buvailo H.I., Makhankova V.G., Kokozay V.N., Babaryk A.A., Omelchenko I.V., Shishkina S.V., Bienko D.C., Jezierska J., Bienko A. (2022). Hybrid Cu-Containing Compounds Based on Lacunary Strandberg Anions: Synthesis under Mild Conditions, Crystal Structure, and Magnetic Properties. Inorg. Chem..

[43] Buvaylo E.A., Kokozay V.N., Makhankova V.G., Melnyk A.K., Korabik M., Witwicki M., Skelton B.W., Vassilyeva O.Y. (2018). Synthesis, Characterization, and Magnetic Properties of a Series of Copper(II) Chloride Complexes of Pyridyliminebenzoic Acids. Eur. J. Inorg. Chem..

[44] Reichardt C. (2003). Solvents and Solvent Effects in Organic Chemistry.

[45] Fuenteabla P., Seron L., Sachez C., Manzur J., Paredes-Garcia V., Pizzaro N., Cepeda M., Venegaz-Yazigi D., Spodine E. (2014). Macrocyclic ZnII and CuII complexes as guests of the hybrid composites based on the layered MnPS3 phase. Comparison of spectroscopic properties. J. Coord. Chem..

[46] Brudenell S.J., Spiccia L., Tiekink E.R.T. (1996). Binuclear Copper(II) Complexes of Bis(pentadentate) Ligands Derived from Alkyl-Bridged Bis(1,4,7-triazacyclonane) Macrocycles. Inorg. Chem..

[47] Chinnaraja E., Arunachalam R., Subramanian P.S. (2016). Enantio- and Diastereoselective Synthesis of b-Nitroalcohol via Henry Reaction Catalyzed by Cu(II), Ni(II), Zn(II) Complexes of Chiral BINIM Ligands. Chem. Select.

[48] Grzybowski J.J., Merrell P.H., Urbach F.L. (1978). Dicopper(II) Complexes with Binucleating Ligands Derived from 2-Hydroxy-5-methylisophthaldehyde and 2-(2-aminoethyl)pyridine. Inorg. Chem..

[49] Roznyatovsky V.V., Borisova N.E., Reshetova M.D., Ustynyuk Y.A., Aleksandrov G.G., Eremenko I., Moiseev I.I. (2004). Dinuclear and polynuclear transition metal complexes with macrocyclic ligands. 6. New dinuclear copper(II) complexes with macrocyclic Schiff bases derived from 4-tert-butyl-2,6-diformylphenol. Russ. Chem. Bull..

[50] Qing S., Qiao-Lin W., Guang-Hua L., Xiao-Ming L., Ying M. (2007). Bis-salicylaldiminato zinc complexes: Syntheses, characterization and luminescent properties. Polyhedron.

[51] Fan J., Autschbach J., Ziegler T. (2010). Electronic Structure and Circular Dichroism of Tris(bipyridyl) Metal Complexes within Density Functional Theory. Inorg. Chem..

[52] Enamullah M., Uddin A.K.M.R., Pescitelli G., Berardozzi R., Makhloufi G., Vasylyeva V., Chamayou A.-C., Janiak C. (2014). Induced chirality-at-metal and diastereoselectivity at Δ/Λ-configured distorted square-planar copper complexes by enantiopure Schiff base ligands: Combined circular dichroism, DFT and X-ray structural studies. Dalton Trans..

[53] Ahmed N., Tripathi S., Sarkar A., Ansari K.U., Das C., Prajesh N., Horike S., Boomishankar R., Shanmugam M. (2020). Chiral tetranuclear copper(II) complexes: Synthesis, optical and magnetic properties. New J. Chem..

[54] Barwiolek M., Jankowska D., Kaczmarek-Kędziera A., Wojtulewski S., Skowroński L., Rerek T., Popielarski P., Muziol T.M. (2022). Experimental and Theoretical Studies of the Optical Properties of the Schiff Bases and Their Materials Obtained from o-Phenylenediamine. Molecules.

[55] Basumallick L., George S.D., Randall D.W., Hedman B., Hodgson K.O., Fujisawa K., Solomon E.I. (2002). Spectroscopic comparison of the five-coordinate [Cu(SMeIm)(HB(3,5-iPr2pz)3)] with the four-coordinate [Cu(SCPh3)(HB(3,5-iPr2pz)3)]: Effect of coordination number increase on a blue copper type site. Inorg. Chim. Acta.

[56] Muzioł T.M., Tereba N., Podgajny R., Kedziera D., Wrzeszcz G. (2019). Solvent-assisted structural conversion involving bimetallic complexes based on the Tris(Oxalato)Ferrate(III) unit with the green blue red crystal color sequence. Dalton Trans..

[57] Muzioł T.M., Tereba N., Podgajny R., Pełka R., Czernia D., Wiśniewski M., Koter S., Wrzeszcz G. (2022). Sorption and Magnetic Properties of Oxalato-Based Trimetallic Open Framework Stabilized by Charge-Assisted Hydrogen Bonds. Int. J. Mol. Sci..

[58] Baker M.L., Mara M.W., Yan J.J., Hodgson K.O., Hedman B., Solomon E.I. (2017). K- and L-edge X-ray Absorption Spectroscopy (XAS) and Resonant Inelastic X-ray Scattering (RIXS) Determination of Differential Orbital Covalency (DOC) of Transition Metal Sites. Coord. Chem. Rev..

[59] Chainani A., Sicot M., Fagot-Revurat Y., Vasseur G., Granet J., Kierren B., Moreau L., Oura M., Yamamoto A., Tokura Y. (2017). Evidence for Weakly Correlated Oxygen Holes in the Highest-Tc Cuprate Superconductor HgBa2Ca2Cu3O8+δ. Phys. Rev. Lett..

[60] Pearce C.I., Pattrick R.A.D., Vaughan D.J., Henderson C.M.B., van der Laan G. (2006). Copper oxidation state in chalcopyrite: Mixed Cu d9 and d10 characteristics. Geochim. Cosmochim. Acta.

[61] Wang H., Friedrich S., Li L., Mao Z., Ge P., Balasubramanian M., Patil D.S. (2018). L-edge sum rule analysis on 3d transition metal sites: From d10 to d0 and towards application to extremely dilute metallo-enzymes. Phys. Chem. Chem. Phys..

[62] Pattrick R.A.D., Coker V.S., Pearce C.I. (2008). The oxidation state of copper and cobalt in carrollite, CuCo_2_S_4_. Can. Mineral..

[63] Sarangi R., Aboelella N., Fujisawa K., Tolman W.B., Hedman B., Hodgson K.O., Solomon E. (2006). X-ray Absorption Edge Spectroscopy and Computational Studies on LCuO2 Species:  Superoxide−CuII versus Peroxide−CuIII Bonding. J. Am. Chem. Soc..

[64] George S.D., Metz M., Szilagyi R.K., Wang H., Cramer S.P., Lu Y., Tolman W.B., Hedman B., Hodgson K.O., Solomon E.I. (2001). A Quantitative Description of the Ground-State Wave Function of CuA by X-ray Absorption Spectroscopy: Comparison to Plastocyanin and Relevance to Electron Transfer. J. Am. Chem. Soc..

[65] Oxford Diffraction Ltd. (2000). CrysAlis RED and CrysAlis CCD.

[66] Kabsch W. (2010). XDS. Acta Cryst..

[67] Krug M., Weiss M.S., Heinemann U., Mueller U. (2012). XDSAPP: A graphical user interface for the convenient processing of diffraction data using XDS. J. Appl. Crystallogr..

[68] Walker N., Stuart D. (1983). An empirical method for correcting diffractometer data for absorption effects. Acta. Cryst..

[69] Sheldrick G.M. (2008). A short history of SHELX. Acta Crystallogr. Sect. A.

[70] Brandenburg K. (2001). DIAMOND, release 2.1e.

[71] Farrugia L.J.J. (1997). ORTEP-3 for Windows—A version of ORTEP-III with a Graphical User Interface (GUI). Appl. Crystallogr..

[72] Bain G.A., Berry J.F. (2008). Diamagnetic corrections and Pascal’s constants. J. Chem. Educ..

[73] Stoll S., Schweiger A. (2006). EasySpin, a Comprehensive Software Package for Spectral Simulation and Analysis in EPR. J. Magn. Reson..

[74] Stoll S. (2015). CW-EPR Spectral Simulations: Solid State. Methods Enzymol..

[75] Frisch M.J., Trucks G.W., Schlegel H.B., Scuseria G.E., Robb M.A., Cheeseman J.R., Scalmani G., Barone V., Petersson G.A., Nakatsuji H. (2016). Gaussian 16, revision B.01.

[76] David G., Wennmohs F., Neese F., Ferre N. (2018). Chemical Tuning of Magnetic Exchange Couplings Using Broken-Symmetry Density Functional Theory. Inorg. Chem..

[77] Bencini A., Gatteschi D.X. (1980). Alpha.-SW calculations of the electronic structure and magnetic properties of weakly coupled transition-metal clusters. The [Cu_2_Cl_6_]_2_-dimers. J. Am. Chem. Soc..

[78] Neese F., Wennmohs F., Becker U., Riplinger C. (2020). The ORCA quantum chemistry program package. J. Chem. Phys..

[79] Ravel B., Newville M. (2005). ATHENA, ARTEMIS, HEPHAESTUS: Data analysis for x-ray absorption spectroscopy using IFEFFIT. J. Synchrotron Radiat..

[80] Mueller U., Förster R., Hellmig M., Huschmann F.U., Kastner A., Malecki P., Pühringer S., Röwer M., Sparta K., Steffien M. (2015). The macromolecular crystallography beamlines at BESSY II of the helmholtz-zentrum Berlin: Current status and perspectives. Eur. Phys. J. Plus.

