# Supplementary material for: New Dinuclear Macrocyclic Copper(II) Complexes as Potentially Fluorescent and Magnetic Materials
Source: Int J Mol Sci. 2023 Feb 3;24(3):3017. doi: 10.3390/ijms24033017 (PMC9918273; doi:10.3390/ijms24033017)
Supplement: Supplementary file 1 [file ijms-24-03017-s001.zip › ijms-2165332-supplementary.pdf]

New dinuclear macrocyclic copper(II) complexes as potentially fluorescent and magnetic materials

Table of contents:

Figure S1. IR spectrum of **K1**.

Figure S2. IR spectrum of **K2**.

Figure S3. a) TG-DTA traces of **K1** and b) **K2.**

Figure S4. a) TG-DTA traces of **K1 a**nd b) IR spectra decomposition products.

Figure S5. a) TG-DTA traces of **K2** and b) IR spectra decomposition products.

Figure S6. XRD of the thermal analysis residue of **K1**.

Figure S7. XRD of the thermal analysis residue of **K2**.

Figure S8. Powder EPR spectra recorded for **K1** (A) and **K2** (B) at 77 K.

Figure S9. Powder EPR spectra recorded for **K1** and **K2** at 350 K.

Figure S10. SEM/EDX images of **K1** mapping scanning size 300 μm.

Figure S11. SEM/EDX images of **K2** mapping scanning size 300 μm.

Figure S12. AFM of **K1**/Si, thermal deposition, scan size 5 μm, R_a=4.02 nm, R_q=5.36 nm.

Figure 13. IR DRIFT of **K1**/Si and **K2**/Si materials- thermal vapour deposition.

Table S1. The theoretical values of the $J(Cu_1-Cu_2)$ magnetic coupling constants [cm^{-1}], estimated within the broken-symmetry and spin-flip approach with B3LYP functional and def2-SVP/def2-QZVPP(Cu) basis set according to the three schemes: spin-projected (SP), spin-unprojected (SUP) and approximate spin-projected (AP).

Table S2. Relevant photophysical data of studied compounds: ë$_{em}$, ë$_{ex}$ [nm] Bp=8; λ[nm] (ε [dm^3 mol^{-1} cm^{-1}]); Stoke shift [nm], [cm^{-1}]; Q Bp=5.

Table S3. Fluorescence data of the thin materials **K1**/Si and **K2**/Si obtained by thermal vapour deposition.

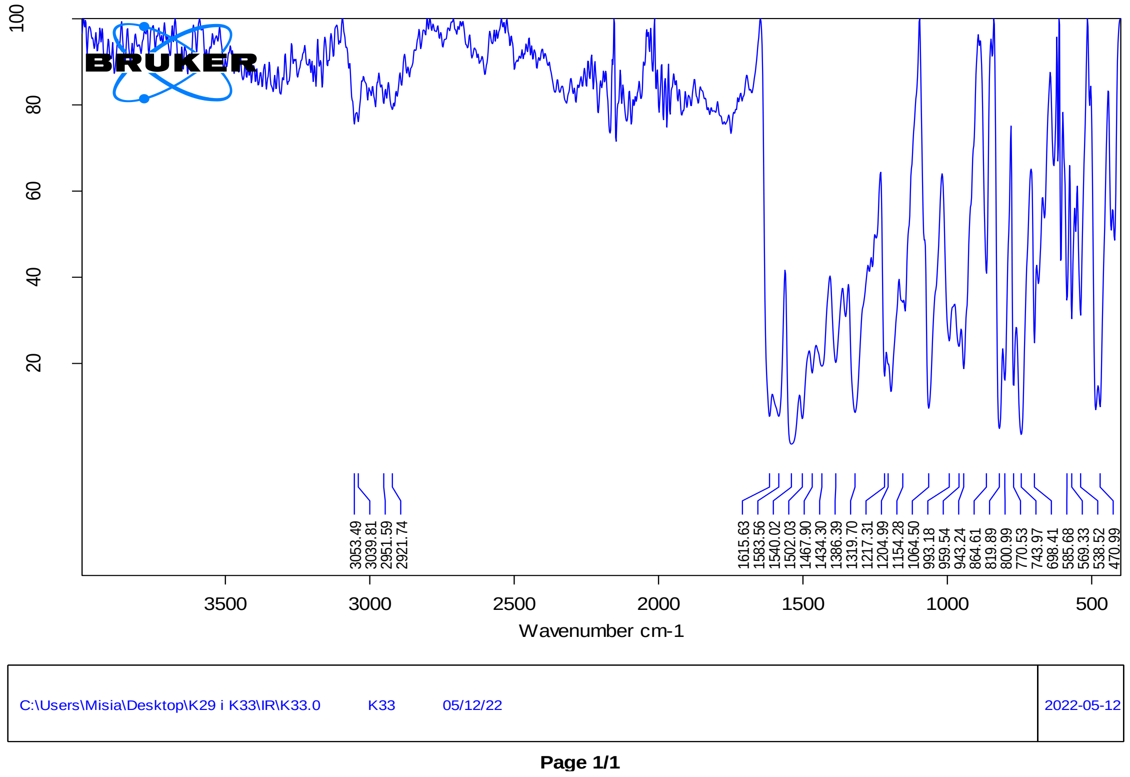

Figure S1. IR spectrum of **K1**.

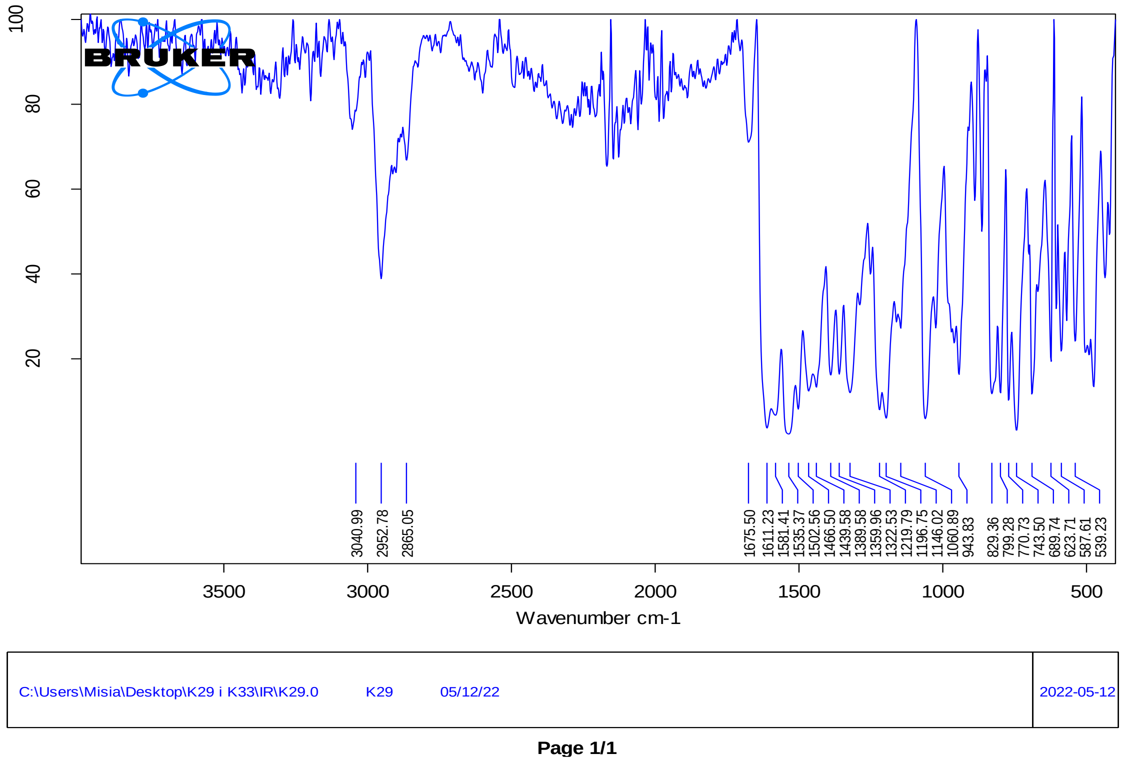

Figure S2. IR spectrum of **K2**.

Thermal analysis

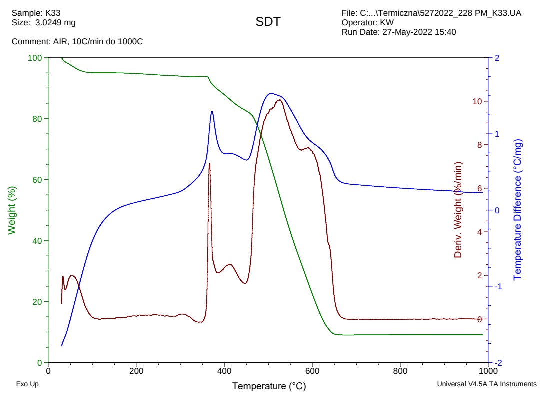

a)

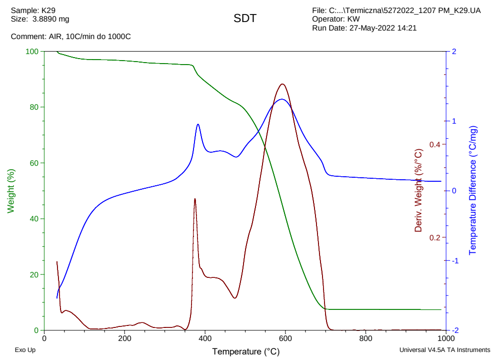

b)

Figure S3. **a)** TG-DTA traces of **K1** and **b) K2.**

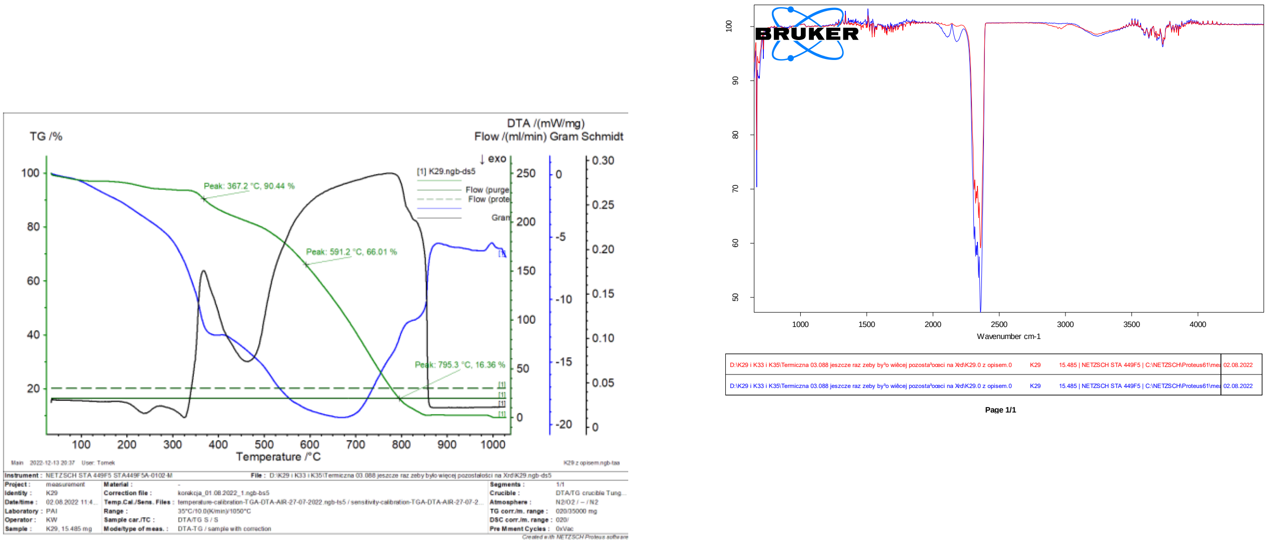

a) b)

Figure 4. a) TG-DTA traces of **K1** and **b)** IR spectra decomposition product

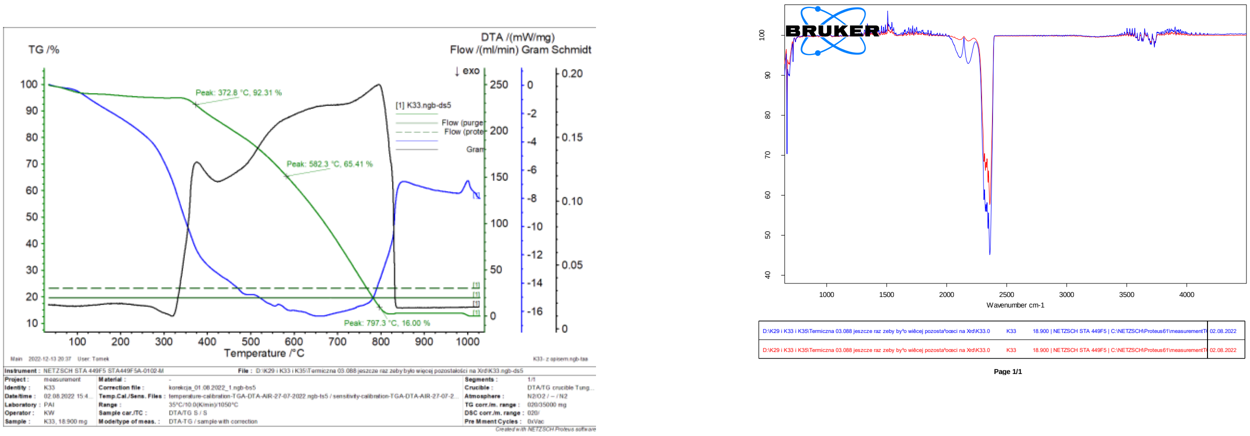

a) b)

Figure S5. **a)** TG-DTA traces of **K2** and **b)** IR spectra decomposition products.

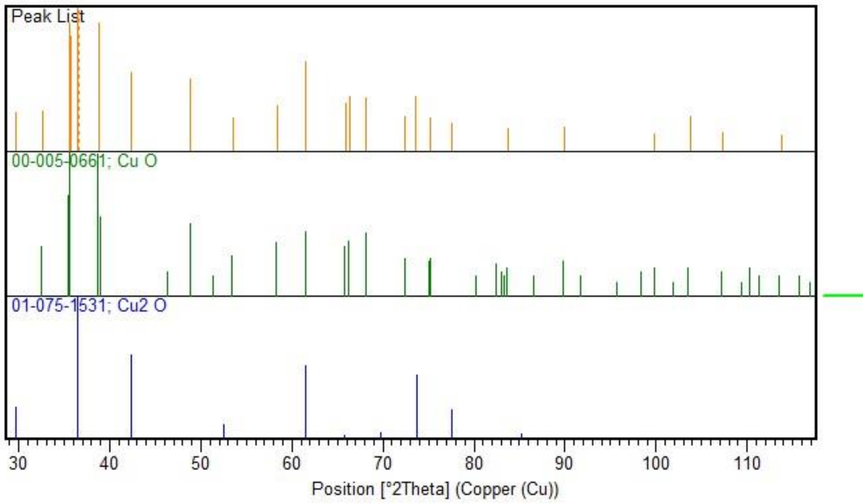

Figure S6. XRD of the thermal analysis residue of **K1**.

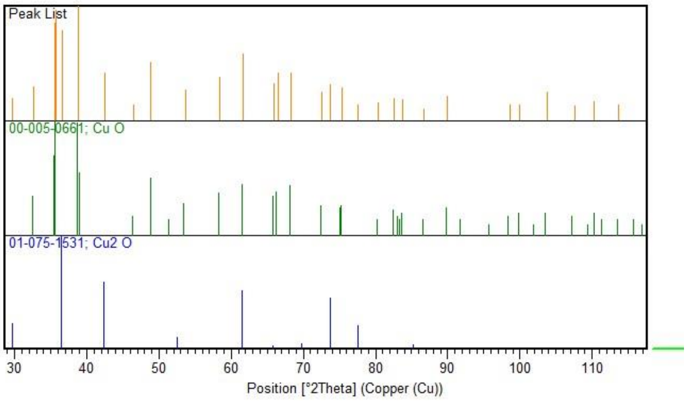

Figure S7. XRD of the thermal analysis residue of **K2**.

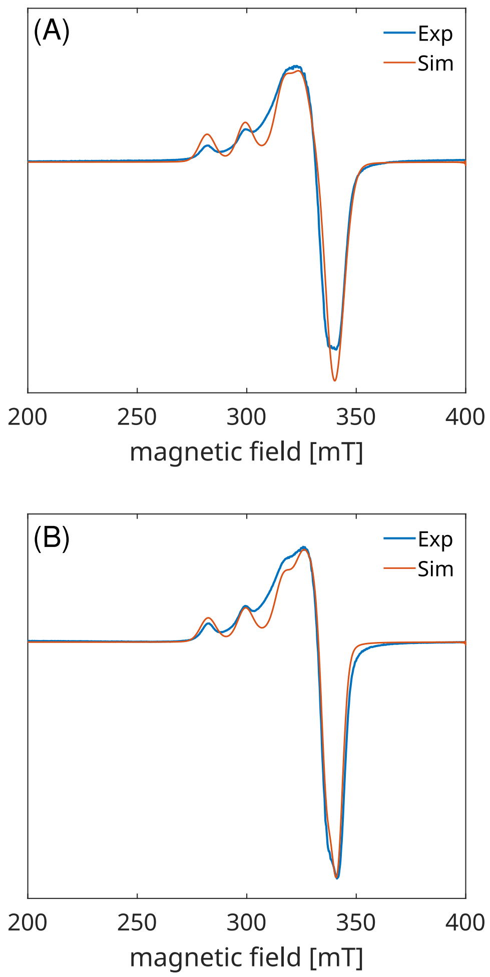

Figure S8. Powder EPR spectra recorded for **K1** (A) and **K2** (B) at 77 K.

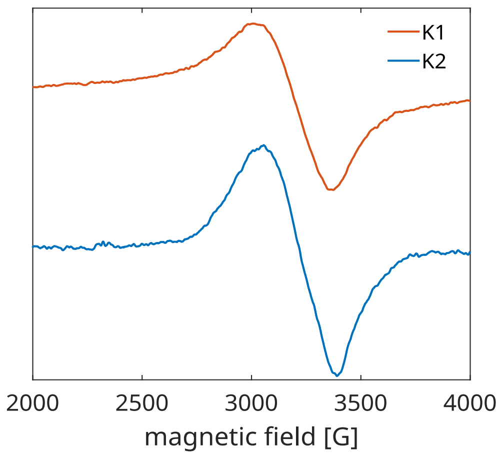

Figure S9. Powder EPR spectra recorded for **K1** and **K2** at 350 K.

Table S1. The theoretical values of the J(Cu_1-Cu_2) magnetic coupling constants [cm^{-1}], estimated within the broken-symmetry and spin-flip approach with B3LYP functional and def2-SVP/def2-QZVPP(Cu) basis set according to the three schemes: spin-projected (SP), spin-unprojected (SUP) and approximate spin-projected (AP).

	**K1**	**K2**
	**Broken Symmetry/B3LYP/def2-SVP+def2-QZVPP for Cu**	
E(High-Spin)-E(BrokenSym)	673.941 (antiferromagnetic)	693.172 (antiferromagnetic)
J(SP)	-673.94	-693.17
J(SUP)	-336.97	-346.59
J(AP)	-632.18	-649.28
	**Spin-Flip/B3LYP/def2-SVP+def2-QZVPP for Cu**	
E(High-Spin)-E(BrokenSym)	674.353 (antiferromagnetic)	693.295 (antiferromagnetic)
J(SP)	-674.35	-693.29
J(SUP)	-337.18	-346.65
J(AP)	-632.58	-649.42

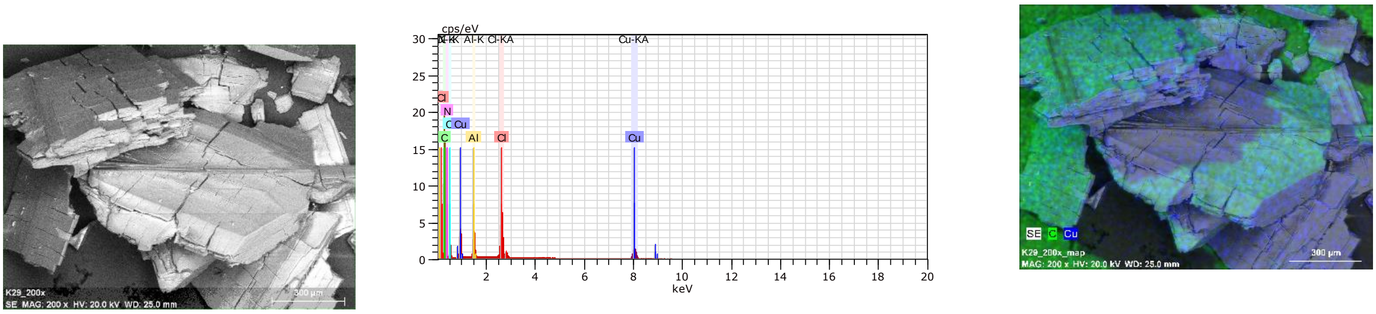

Figure S10. SEM/EDX images of **K1** maping scanning size 300 μm.

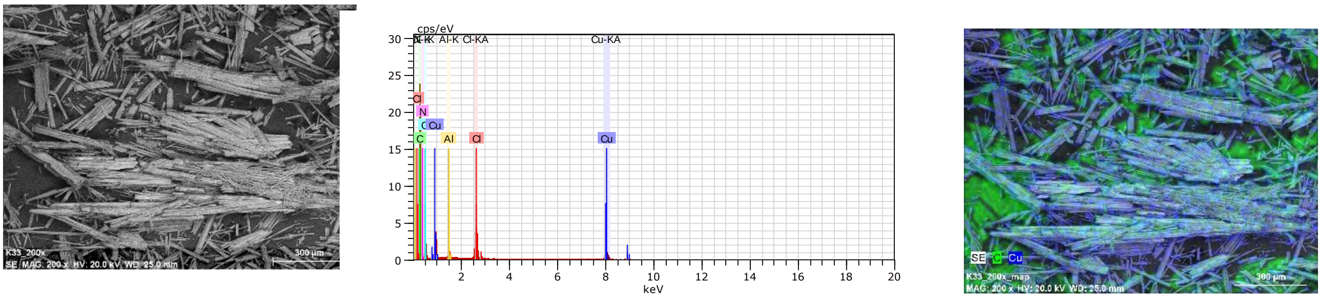

Figure S11. SEM/EDX images of **K2** maping scanning size 300 μm.

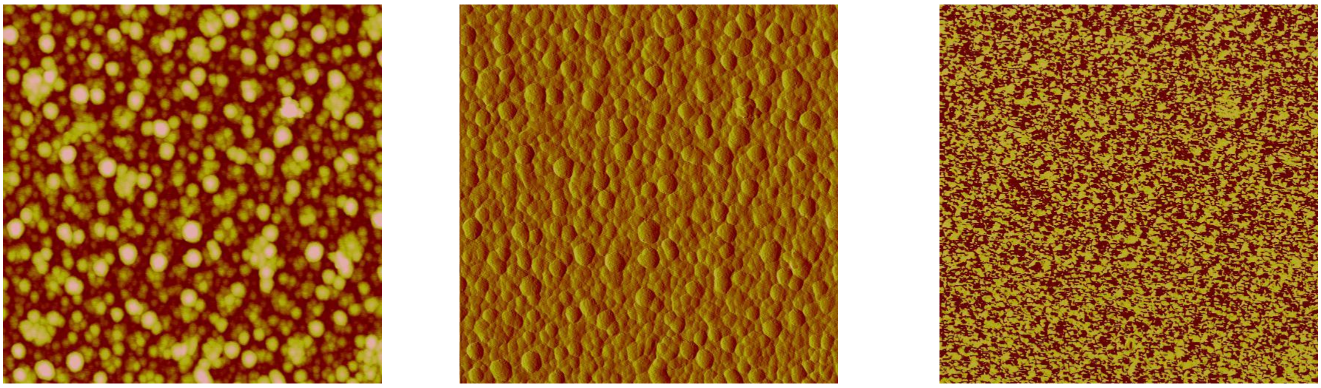

Height **Amplitude** **Phase**

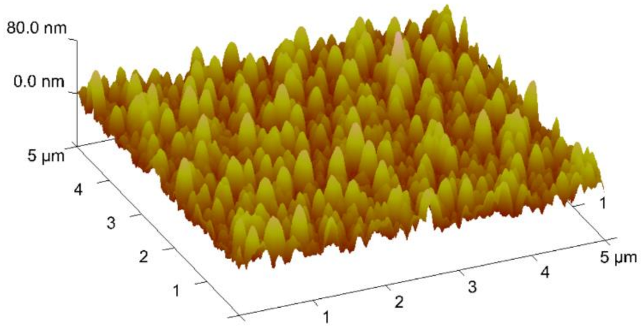

Figure S12. AFM of **K1**/Si, thermal deposition, scan size 5 µm, R_a=4.02 nm, R_q=5.36 nm.

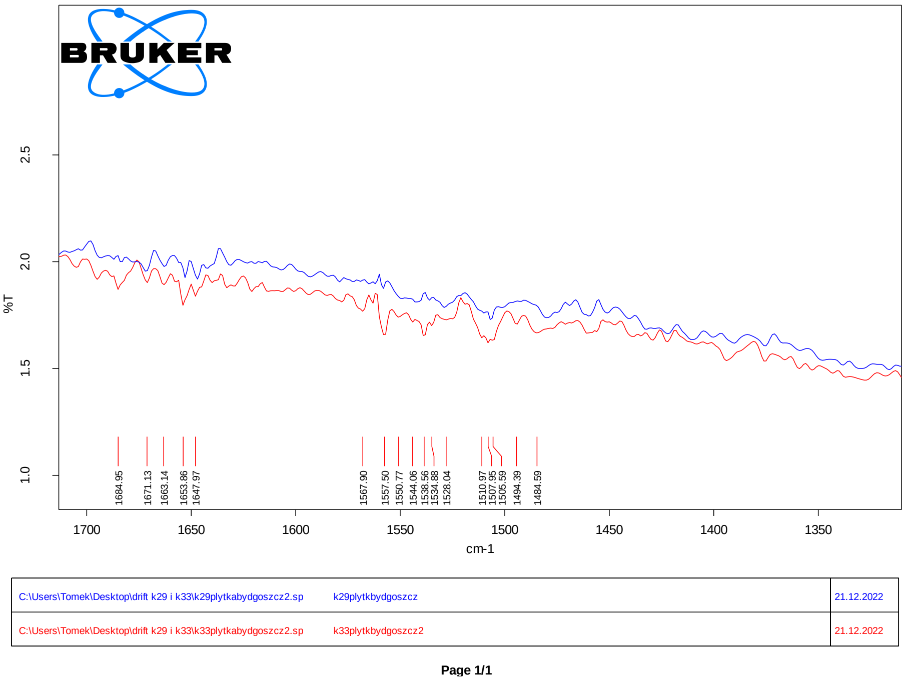

Figure 13. IR DRIFT of **K1**/Si and **K2**/Si materials- thermal vapour deposition.

Table S2. Relevant photophysical data of studied compounds: ë$_{em}$, ë$_{ex}$ [nm] Bp=8; λ[nm] (ε [dm^3 mol^{-1} cm^{-1}]); Stoke shift [nm], [cm^{-1}]; Q Bp=5.

Comp.	Solvent	λ_{ex} [nm]	λ_{em} [nm]	Fluoresc. Intensity a. u.	λ[nm] (ε [dm^3 mol^{-1} cm^{-1}])	A	Q	Stoke shift [nm]	Stoke shift [cm^{-1}]
K1	acetone	350	**409**	1688725	**402** (39628)	**0,128**	0,027	**7**	**1428571**
	methanol		**412**	1151660	**388** (43963); 324 (46440) sh; 274 (104025)	**0,142**; 0,150; 0,336	0,013	**24**	**416667**
	chloroform		**398**	201250	**408** (37461); 324 (40248) sh; 272 (114861)	**0,121**; 0,130; 0,371	0,0019	**10**	**1000000**
					660 (13313)	**0,043**			
	DMSO		**424**	3021975	**398** (44582); 290 (130960) sh	**0,144**; 0,423	0,017	**26**	**384615**
K2	acetone	350	**408**	1315030	**400** (40867)	**0,132**	0,035	**8**	**1250000**
	methanol		**420**	438415	**388** (49845); 326 (50774) sh; 274 (123220)	**0,161**; 0,164; 0,398	0,011	**32**	**312500**
	chloroform		**392**	260640	**404** (43344); 328 (42724) sh; 272 (124458)	**0,140**; 0,138; 0,402	0,003	**12**	**833333**
					664 (12384)	**0,040**			
	DMSO		**424**	1934125	**394** (45201)	**0,146**	0,021	**30**	**333333**
	acetonitrile		**409**	982115	**392** (46749)	**0,151**	0,038	**17**	**588235**

Table S3. Fluorescence data of the thin materials **K1**/Si and **K2**/Si – thermal vapour deposition.

Comp.	λ_{ex} [nm]	λ_{em} [nm]	Fluoresc. Intensity a. u.
	250	318	11715
		390	13580
		531	7320
**K2**	320	386	27980
		481	15870
		575	5050
		686	9145
	250	326	367045
		377	271315
		472	68955
**K1**	300	376	414115
		472	89570
	320	372	518885
		474	107320
